# Moth flies and sand flies (Diptera: Psychodidae) in Cretaceous Burmese amber

**DOI:** 10.7717/peerj.1254

**Published:** 2015-09-17

**Authors:** Frauke Stebner, Mónica M. Solórzano Kraemer, Sergio Ibáñez-Bernal, Rüdiger Wagner

**Affiliations:** 1 Steinmann-Institut, Abteilung Paläontologie, Bonn, Germany; 2 Senckenberg Forschungsinstitut und Naturmuseum, Frankfurt am Main, Germany; 3 Instituto de Ecología, A. C. Red Ambiente y Sustentabilidad, Xalapa, Veracruz, México; 4 FB 10 Naturwissenschaften, Institut für Biologie, Kassel, Germany

**Keywords:** Psychodidae, New genera, Cretaceous, Burmese amber, New subfamily, New species

## Abstract

One new subfamily, four new genera and 10 new species of Psychodidae are described from Burmese amber which significantly increases our knowledge about this group in the Cretaceous. Protopsychodinae n. subfam. probably represents the oldest known ancestor of modern Psychodinae and includes three species within two genera: *Datzia setosa* gen. et sp. n., *Datzia bispina* gen. et sp. n., and *Mandalayia beumersorum* gen. et sp. n. Sycoracinae and Phlebotominae are represented by two genera each in the studied material, *Palaeoparasycorax globosus* gen. et sp. n., *Palaeoparasycorax suppus* gen. et sp. n., *Parasycorax simplex* sp. n., and *Phlebotomites aphoe* sp. n. and *Phlebotomus vetus* sp. n., respectively. Bruchomyiinae is represented by *Nemopalpus quadrispiculatus* sp. n. Furthermore, one genus of an incertae sedis subfamily, *Bamara groehni* gen. et sp. n., is described. The systematic positions of the new taxa are discussed.

## Introduction

Psychodidae is a cosmopolitan family with more than 3,000 extant described species (Curler & Moulton, 2012; Ježek & Barták, 2000) which is usually best known to contain hematophagous sand flies, important vectors of diseases. It is considered one of the oldest families of Diptera according to the available fossil records which date back to the Late Jurassic (Ansorge, 1994; see also Grimaldi & Engel, 2005), or maybe even Late Triassic (Blagoderov, Grimaldi & Fraser, 2007).

To date, the classificatory arrangement and phylogenetic relationships to other families and within the family remains a matter of debate (Wagner, 2006; Wagner & Ibáñez-Bernal, 2009). Psychodidae have been intimately related to Trichoceridae, Perissommatidae, Anisopididae, Scatopsidae and Canthyloscelidae, which constitute the infraorder Psychodomorpha (Wood & Borkent, 1989) based on the shape of the larval premandible and mandible which are unique for these families (Woodley, Borkent & Wheeler, 2009). Oosterbroek & Courtney (1995) proposed Tipulidae as the sister group of Trichoceridae within an assemblage including Anisopodidae and Brachycera; nevertheless, as commented by Woodley, Borkent & Wheeler (2009) this conclusion is based on character states that are of questionable quality. Sinclair, Borkent & Wood (2007) suggested that Blephariceromorpha + Psychodomorpha + Brachycera form a monophyletic assemblage. Using data from 18S Ribosomal RNA and both ribosomal (28S rDNA) and protein-coding (CAD, TPI and PGD) genes, Leathers & Judd (2002) and Bertone, Courtney & Wiegmann (2008) respectively discovered that Tanyderidae and Psychodidae are strongly supported as a monophyletic group. These results conflict with current classifications that place Tanyderidae as the sister group of Ptychopteridae (Infraorder Ptychopteromorpha), and so Ptychopteridae is supported as the sister group to the clade of (Tipulomorpha + (Tanyderidae + Psychodidae) + (Bibionomorpha + Brachycera)). In a recent molecular phylogenetic analysis by Curler & Moulton (2012), tanyderids (represented by *Protoplasa fitchii*) showed closest relationship with the psychodid subfamily Sycoracinae (represented by *Sycorax*) as compared with another psychodid subfamily (Horaiellinae, exemplified by *Horaiella*). Krzeminski & Krzeminska (2003) discussed the evolution of four lineages of Diptera in the Triassic, with Diarchineura diversifying since Lower/Middle Triassic being represented by Grauvogeliidae, Kuperwoodidae, Hennigmatidae, Nadipteridae and the recent Tanyderidae and Psychodidae, and Neoneura whose representatives are not known before Upper Triassic. Inside Diarchineura, characterized by five radial veins reaching the wing margin, Psychodomorpha was strongly considered to be related to Tanyderomorpha, Nadipteromorpha, Hennigmatomorpha and Grauvogeliomorpha. Actually, fossil Tanyderidae and Psychodidae are very similar (Ansorge, 1994; Grimaldi & Engel, 2005), so that confusion concerning the correct assignment of fossil taxa has occurred (Woodley, 2005).

**Figure 1:**
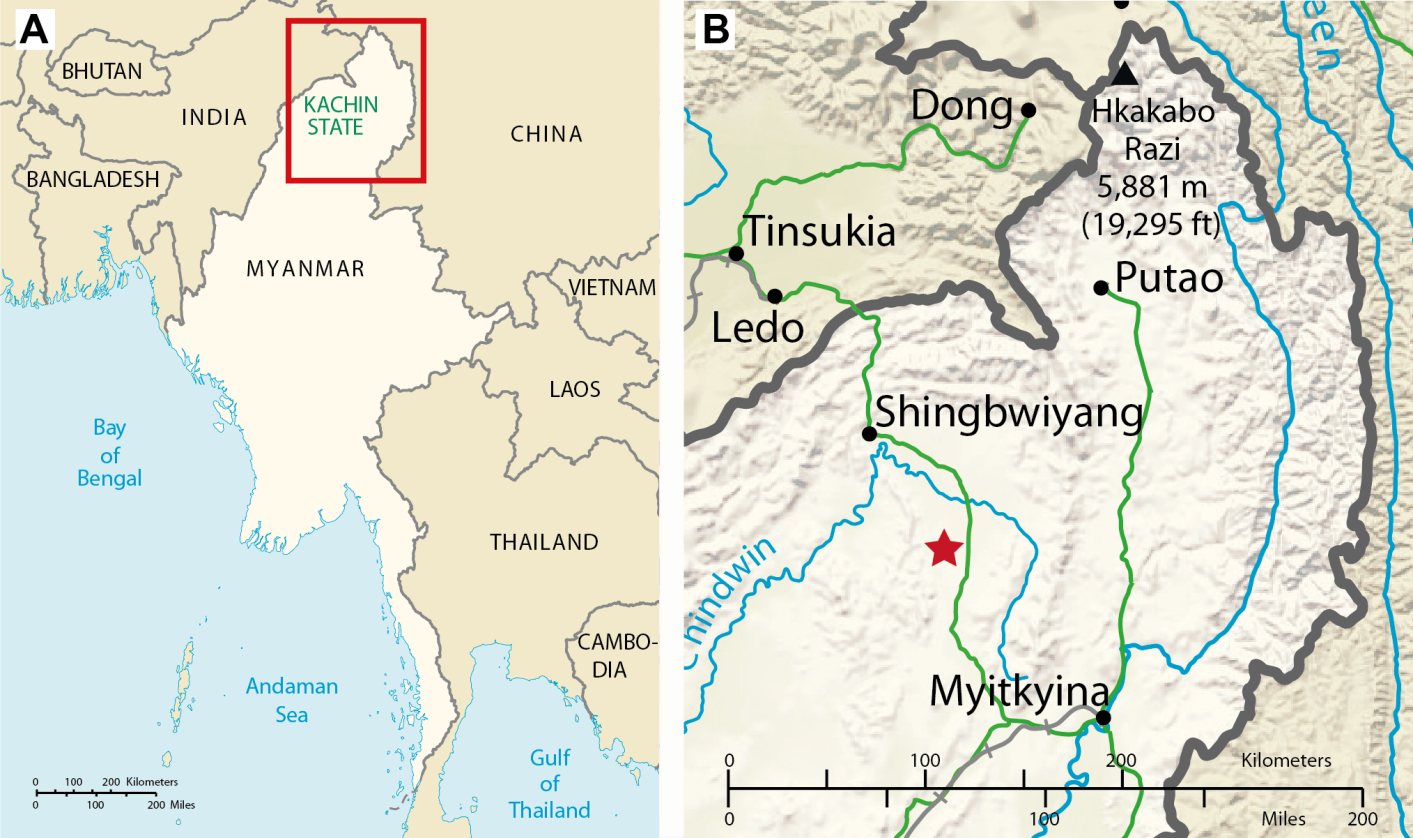
Geographic maps. (A) Map of Myanmar. (B) Position of the amber outcrop in Kachin state (position of outcrop as shown by red asterisk).

Within the family Psychodidae, the classification and phylogenetic relationships are not in better condition. During the history of study, there had been some intentions to separate the group into at least two families (e.g., Adler & Theodor, 1929; Rohdendorf, 1964; Perfil’ev, 1966; Lewis, 1971; Abonnenc, 1972; Williams, 1993; Krzeminski & Krzeminska, 2003) but reasons to do so were more practical for medical entomologists than based on phylogenetic relationships. Currently and in general, specialists recognize six psychodid subfamilies: Horaiellinae, Sycoracinae, Trichomyiinae, Bruchomyiinae, Phlebotominae and Psychodinae (Duckhouse, 1973; Wagner & Ibáñez-Bernal, 2009; Curler & Moulton, 2012), grouped together by two synapomorphies: (1) antennal flagellomeres with membranous sensillae (ascoids, in fact translucid sensillae), and (2) costal wing vein with one or more breaks near base (Wood & Borkent, 1989). Phylogenetic relationships of psychodid subfamilies are still a matter of controversy, probably due to the incorrect interpretation of morphological homologies between them (Curler & Moulton, 2012). The fossil record of Psychodidae is extremely important for helping to trace the significant morphologic homologies, patterns of diversification, and phylogenetic relationships. The fossil record of Psychodidae is moderately rich (Evenhuis, 1994, EDNA Fossil Insect Database, accessed on July 2014: http://edna.palass-hosting.org, The Paleobiology Database, accessed on August 2015: http://fossilworks.org), with most species known from Cenozoic ambers. Some of the oldest amber Psychodidae are from the Lower Cretaceous Lebanese amber (Azar et al., 1999; Azar & Nel, 2003; Azar et al., 2003) and the Lower Cenomanian French amber (Azar, Adaymeh & Jreich, 2007; Lak et al., 2008) (see Table 1). Burmese amber which has been dated on earliest Cenomanian with a minimum age of 98.79 ± 0.62 million years (Shi et al., 2012) derives from Hukawng Valley of Kachin State in northern Myanmar (see Fig. 1) (Cruickshank & Ko, 2003). Although Psychodidae is one of the dominant Diptera groups in this amber (Grimaldi, Engel & Nascimbene, 2002) to date only 9 adult fossils and 2 larvae have been formally described (Duckhouse, 2000; Poinar, 2004; Poinar & Brown, 2004; Poinar, Jacobson & Eisenberger, 2006; Lak et al., 2008; Wagner & Stuckenberg, 2012; Ain Malak, Salamé & Azar, 2013; Azar et al., 2015) (Table 1). Here, 10 further species are described from Burmese amber, implying that the family was already well diversified during the Cretaceous.

**Table 1:** Psychodidae in Cretaceous ambers.

Species	Author	Age
**New Jersey amber**		
*Xenotrichomyia newjerseyiensis*	Azar, Mouawad & Salamé, 2015	Upper Cretaceous
**Amber of Taimyr (Siberia, Russia)**		
*Paleopsychoda zherikhini*	Azar, Adaymeh & Jreich, 2007	Mid-Cretaceous
**French amber**		
*Eophlebotomus carentonensis*	Azar et al., 2003	Lower Cretaceous
*Sycorax neli*	Azar, Tahchy & Perrichot, 2007	Lower Cretaceous
*Trichomyia lengleti*	Lak et al., 2008	Lower Cretaceous
**Burmese amber**		
*Axenotrichomyia boisteli*	Azar et al., 2015	Lower Cretaceous
*Dacochile microsoma*	Poinar & Brown, 2005	Lower Cretaceous
*Eophlebotomus connectens*	(Cockerell, 1920) Duckhouse, 2000	Lower Cretaceous
*Nemopalpus velteni*	Wagner & Stuckenberg, 2012	Lower Cretaceous
*Palaeomyia burmitis*	Poinar, 2004	Lower Cretaceous
*Phlebotomites burmaticus*	Ain Malak, Salamé & Azar, 2013	Lower Cretaceous
*Phlebotomites grimaldii*	Ain Malak, Salamé & Azar, 2013	Lower Cretaceous
*Phlebotomites neli*	Ain Malak, Salamé & Azar, 2013	Lower Cretaceous
Sycoracinae incertae sedis (*Trichomyia swinhoei*)	(Cockerell, 1917) Lak et al., 2008	Lower Cretaceous
2 sand fly larvae	Poinar, Jacobson & Eisenberger, 2006	Lower Cretaceous
**Lebanese amber**		
*Cretapsychoda inexpectata*	Azar et al., 1999	Lower Cretaceous
*Eophlebotomus gezei*	Azar et al., 2003	Lower Cretaceous
*Libanopsychoda abillamai*	Azar et al., 1999	Lower Cretaceous
*Libanophlebotomus lutfallahi*	Azar et al., 1999	Lower Cretaceous
*Mesophlebotomites henningi*	Azar et al., 1999	Lower Cretaceous
*Paleopsychoda inexpectata*	Azar et al., 1999	Lower Cretaceous
*Paleopsychoda jacquelinea*	Azar et al., 1999	Lower Cretaceous
*Paleopsychoda solignaci*	Azar et al., 1999	Lower Cretaceous
*Phlebotomites brevifilis*	Hennig, 1972	Lower Cretaceous
*Phlebotomites longifilis*	Hennig, 1972	Lower Cretaceous
*Protopsychoda hammanaensis*	Azar et al., 1999	Lower Cretaceous
*Protopsychoda nadiae*	Azar et al., 1999	Lower Cretaceous
*Xenopsychoda harbi*	Azar & Ziadé, 2005	Lower Cretaceous

## Material and Methods

A total of 26 fossil psychodid specimens from Burmese amber have been studied. The amber samples were loaned from the public collection of the Senckenberg Forschungsinstitut und Naturmuseum, Frankfurt, Germany (labelled SMF), and the private collection of Carsten Gröhn, Glinde, Germany (labelled Gröhn). Holotypes of the Gröhn collection are deposited in the Geologisch-Paläontologisches Institut der Universität Hamburg, Germany (labelled GPIH), listed under a GPIH number and an additional Gröhn number. Inventory numbers and specimen numbers are listed in Table 2.

**Table 2:** List of investigated specimens of Psychodidae in Burmese amber.

Collection	Inventory no.	Subfamily	Genus	Sex	Species
SMF	Be 2376	Protopsychodinae	*Datzia*	♂(6)	*Datzia bispina*, 6 Paratypes
SMF	Be 2379	Protopsychodinae	*Datzia*	♂(8)	*Datzia bispina*, Holotype + 7 Paratypes
SMF	Be 2380.1	Protopsychodinae	*Datzia*	♂	*Datzia setosa*, Holotype
SMF	Be 2381.1	Protopsychodinae	*Datzia* sp.	♀	
SMF	Be 2383	Incertae sedis	*Bamara* sp.	♀	
SMF	Be 2385.1	Phlebotominae	*Phlebotomites*	♂	*Phlebotomites aphoe*, Holotype
SMF	Be 2385.2	Protopsychodinae	*Mandalayia*	♂	*Mandalayia beumersorum*, Holotype
SMF	Be 2386	Sycoracinae	*Palaeoparasycorax*	♂	*Palaeoparasycorax globosus*, Paratype
SMF	Be 2389	Sycoracinae	*Palaeoparasycorax*	♂	*Palaeoparasycorax globosus*, Holotype
SMF	Be 2390	Bruchomyiinae	*Nemopalpus*	♂	*Nemopalpus quadrispiculatus*, Holotype
SMF	Be 2533	Phlebotominae	*Phlebotomus*	♂	*Phlebotomus vetus,* Holotype
GPIH no. 4417	Gröhn no. 11006	Incertae sedis	*Bamara*	♂	*Bamara groehni*, Holotype
GPIH no. 4418	Gröhn no. 11006	Sycoracinae	*Palaeoparasycorax*	♂	*Palaeoparasycorax suppus*, Holotype
GPIH no. 4449	Gröhn no. 11020	Sycoracinae	*Parasycorax*	♂	*Parasycorax simplex,* Holotype

The morphological and wing venation terminology follows McAlpine (1981) and Wagner & Ibáñez-Bernal (2009). The body length is given in mm including head and terminalia (exceptions are noted at the relevant text passage). In wing nomenclature the terms br and bm are used for the basal radial cell and basal medial cell respectively. In male genitalia other than phlebotomoid specimens the term cerci (sing., cercus) is used for oval, setose, probably soft appendages of tergite 9. In non-phlebotomoid taxa, surstyli (sing., surstylus) is used in cases with elongate, rigid, and evidently jointed appendages with strongly differentiated or specialized setae named here as tenacula (sing., tenaculum), that have been referred to as retinacula (sing. retinaculum) by some authors.

All samples from the SMF collection have been ground and polished to pieces of minimal possible size. If possible, pieces with several inclusions were cut and prepared further to achieve an optimal view on the studied specimens. The only exceptions are two pieces containing several males of one species (SMF Be 2379 and SMF Be 2376) which are valuable reports of swarming behavior and were therefore not cut.

For taxonomic identification and descriptions a Leica MZ 12.5 and a MZ 16 were used. Drawings were prepared with the aid of a drawing tube and performed with Adobe Illustrator CS5.1. Measurements are given in millimeters. Photographs of the amber inclusions were made with a Leica MZ 16 Stereomicroscope with a JVC ky-F70B Digital Camera. Compound photographs merging different focal levels to a single image were created by using Discus software equipped with stacking function.

As in many other dipteran families taxonomy of Psychodidae is largely based on male morphology because they show more useful taxonomic characters than females and in many cases it is not possible to align a single female to a male. Therefore descriptions of new taxa are based on males only in the present work.

The electronic version of this article in Portable Document Format (PDF) will represent a published work according to the International Commission on Zoological Nomenclature (ICZN), and hence the new names contained in the electronic version are effectively published under that Code from the electronic edition alone. This published work and the nomenclatural acts it contains have been registered in ZooBank, the online registration system for the ICZN. The ZooBank LSIDs (Life Science Identifiers) can be resolved and the associated information viewed through any standard web browser by appending the LSID to the prefix “http://zoobank.org/”. The LSID for this publication is: urn:lsid:zoobank.org:pub:DEED752E-1A9A-4886-9966-2490D5556EE8. The online version of this work is archived and available from the following digital repositories: PeerJ, PubMed Central and CLOCKSS.

## Systematic Paleontology

**Table:** 

Order: Diptera Linnaeus, 1758
Family: Psychodidae Newman, 1834
Protopsychodinae **n. subfam.**
urn:lsid:zoobank.org:act:568EDF6C-C7B1-457D-AF44-4C4D643C00B6

*Type-genus*: *Datzia***n. gen.**

*Derivation of name: Proto*, Greek preposition (=before) and *Psychodinae*, name of extant subfamily.

*Diagnosis*: Psychodids with round eyes, without or with only very weakly developed eye bridge, antenna with scape, pedicel and 14 short flagellomeres, 1st flagellomere not more than 1.5× the length of 2nd, each flagellomere with cylindrical node and short internode, that could be centric or slightly eccentric, apical flagellomere with apiculus; elongate mouthparts, palpus with 5 segments, the last one elongate; mesonotum with 2 distinct rows of dorsocentral bristles; wing with apex broadly rounded, short petiolate, R with 5 branches, thus 2 veins between radial and medial forks, with cross-veins h, r-m, and M_3_ and CuA_1_ in contact directly or by a very short cross-vein so that cells br and bm are closed, subcosta and anal veins elongate, R_5_ originates distal to the R_2+3_ + R_4_ fork; CuA_2_ and A veins converge distally and terminate in costa close to one another; male terminalia inverted, epandrium large and quadrate with relative long surstyli, gonopodia (i.e., gonocoxite + gonostylus) elongate.

*Remarks*: The subfamily name (Protopsychodinae) is different from the type genus name (*Datzia*) because the genus name *Protopsychoda* already exists. Protopsychodinae have some features in common with the subfamily Psychodinae such as inverted male terminalia, epandrium with the form of a plate and the presence of surstyli. They differ, however in the number of palpal segments, which is 5 in Protopsychodinae, and in having eyes rounded, both characters which are present in the subfamilies Bruchomyiinae and Phlebotominae. Thus the set of the above mentioned characters is unique in Psychodidae and we characterize and describe a new subfamily.

*Datzia* **n. gen.**

urn:lsid:zoobank.org:act:A8D8B8AE-229E-469F-A8F8-CA496B45FFA0

*Type-species*: *Datzia bispina***n. gen.**

*Derivation of name:* Dedicated to the Erika und Walter Datz-Stiftung for giving the opportunity to acquire amber material for the present work.

*Diagnosis*: Rear basal wing margin sinuous, male surstyli with few strong terminal modified claw-like setae or tenacula.

*Datzia bispina* **n. sp.**

urn:lsid:zoobank.org:act:B094011C-331E-487E-8700-B4FB53EEB95C

**Figure 2:**
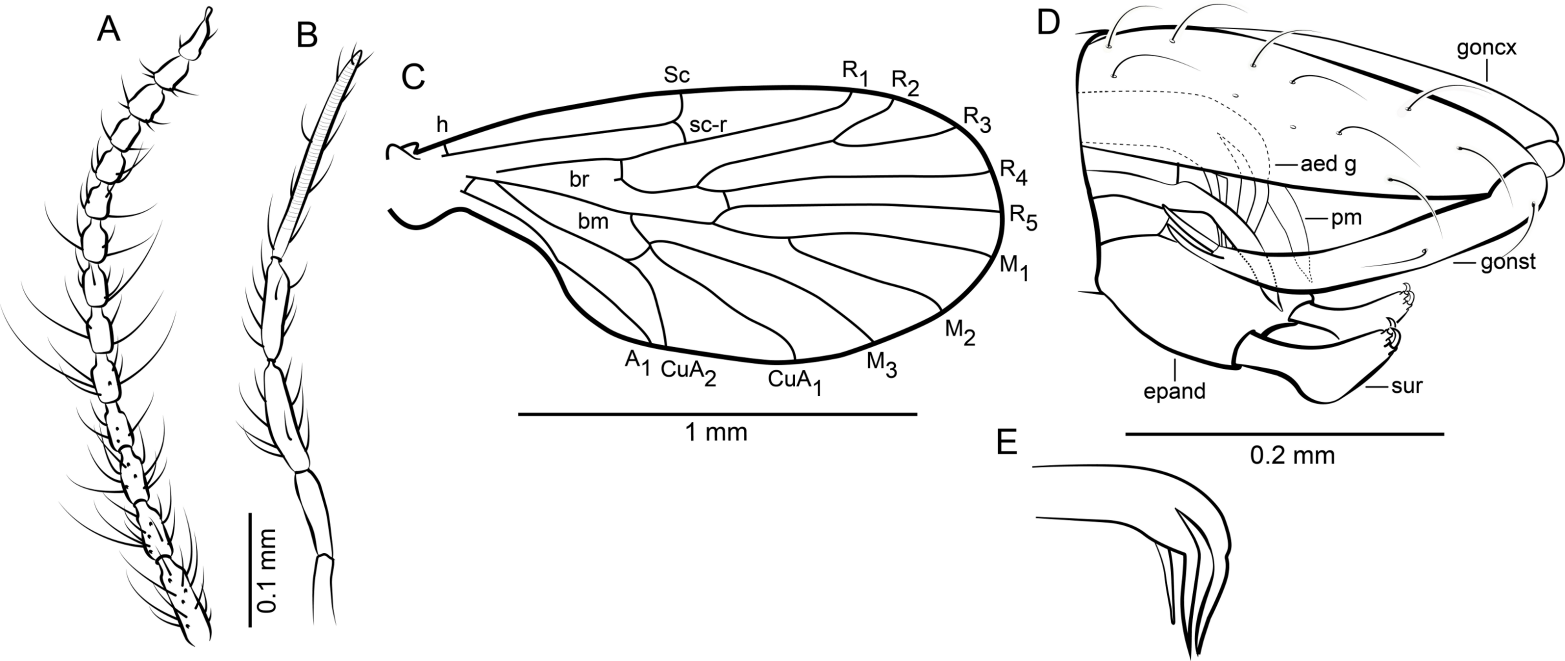
*Datzia bispina* n. sp. ♂. (A) Antenna. (B) Palpus. (C) Wing. (D) Terminalia lateral view. (E) Aedeagus lateral view. Abbreviations: A_1_, first branch of anal vein; aed g, aedeagal guide; bm, basal medial cell; br, basal radial cell; CuA_1_, CuA_2_, branches of anterior branch of cubital vein; epand, epandrium; goncx, gonocoxite; gonst, gonostylus; h, humeral cross-vein; M_1_, M_2_, M_3_, branches of medial vein; pm, paramere; R_1_, R_2_, R_3_, R_4_, R_5_, branches of radial vein; Sc, subcosta; sc-r, subcostal-radial cross-vein; sur, surstylus.

**Figure 3:**
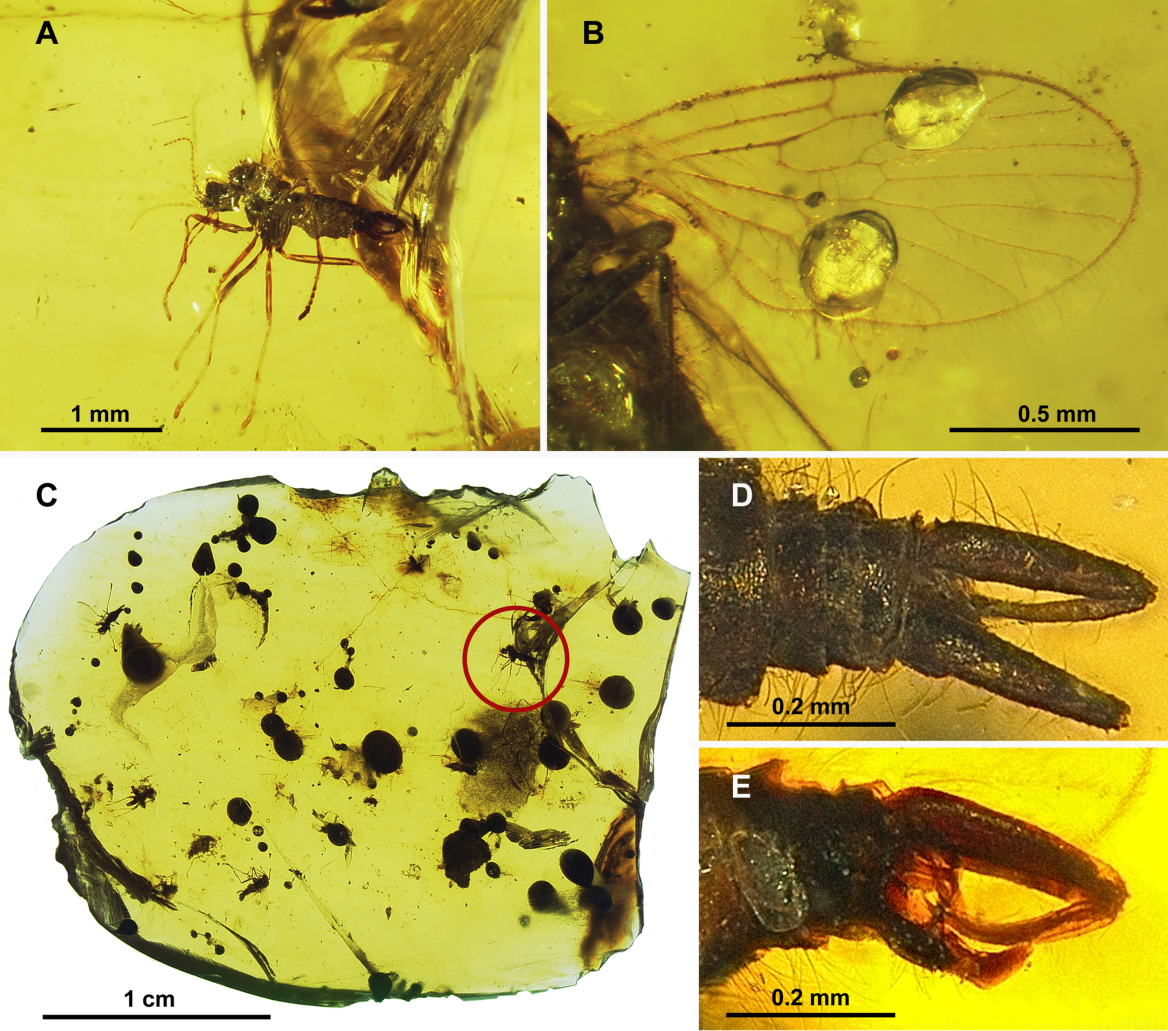
Protopsychodinae n. subfam. (A) *Datzia bispina* n. sp. ♂, Holotype specimen. (B) *Datzia bispina* n. sp. ♂, Holotype: wing. (C) Piece of amber with *Datzia bispina* n. sp., ♂ Holotype (position as shown by red circle) and 7 ♂ Paratypes. (D) *Datzia bispina* n. sp. ♂, Paratype: terminalia dorsal view. E *Datzia bispina* n. sp. ♂ , Holotype: terminalia lateral view.

Figs. 2A–2E and 3A–3E

*Material*: Holotype male SMF Be 2379 (location as shown in Fig. 3C) and 7 male Paratypes; SMF Be 2376 Paratypes 6 males. Syninclusions SMF Be 2379: Diptera, Chironomidae (1); Sternorrhyncha? (2); Thysanoptera (1). Syninclusions SMF Be 2376: Trichoptera (1); Thysanoptera (1); Diptera, Chironomidae (6), Ceratopogonidae? (1); Coleoptera (1).

*Type-locality*: Hukawng Valley of the northern state of Kachin.

*Stratigraphic horizon*: 98.79 ± 0.62 million years, Upper Cretaceous (earliest Cenomanian) (Shi et al., 2012).

*Derivation of name:* Refers to the two modified setae or claw-like tenacula at the apex of surstylus.

*Diagnosis*: A small psychodid species with palpus segment 5 as long as 3 + 4, gonostylus apically bifid, parameres paired and blade shaped, surstyli boot-shaped, apically with two short strong claw-like tenacula.

*Description*: Body length: 1.72 mm.

*Head:* Eyes oval, without eye bridge, but eyes cover great part of the head. Diameter of individual facets about 0.02 mm. Antenna (Fig. 2A) with 16 antennomeres, scape increasing in diameter distally, with some strong distal setae, pedicel elongate oval, and 14 flagellomeres with cylindrical node and short internode. Flagellomere 1 is the longest but not longer than 2 + 3, flagellomeres 1–9 with a short but distinct internode, in the remaining distal segments the internode is slim and very short; apical flagellomere with apiculus. Relative proportions of antennomeres: 10-9-12-9-9-8-8-8-7-7-7-7-6-5-5-8; ascoids unrecognizable. Mouthparts elongate with long thin blade-shaped mandibles, as for biting (even in males). Maxillae and labrum elongate as well. Palpus (Fig. 2B) five segmented, relative proportions of palpomeres: 10-14-17-14-32, segment 5 soft and with secondary annulations. *Thorax*: Mesonotum covered with long hairs and setae, and 2 rows with at least 6 dorsocentral bristles each. Tibia with long bristles, tarsomeres cylindrical, tarsal segment 4 beveled.

*Wing*: (Fig. 2C) Broad, scarcely setose, hairs on wing veins and along costal vein, longest in the anal area. Sc long, ending in costa at approximately right angle apical to the origin of Rs, with sc-r cross-vein. Humeral vein obscured in holotype but clearly visible in other paratype specimens present in the same piece of amber and therefore shown in Fig. 2E. Radius with 5 veins, stem of radial fork 1.5 times longer than R_2_. R_5_ ending in wing tip, and with basal cross-vein towards stem of M_1+2_. Basal part of Rs with a short peduncle into cell br. Media with 3 veins; M_3_ and CuA_1_ originate at close quarters at the tip of cell bm. Anal vein exceptionally long and almost straight except for a median discontinuity, terminating in costa close to CuA_2_. Hind margin of wing basis slightly convex so the anal lobe is accentuated, then costa curved so that the wing appears petiolate. Cells br and bm closed. Wing length 1.49 mm.

*Abdomen*: With eight visible segments and inverted terminalia. Inversion of terminalia by segment 9, a basal ring is clearly visible. Terminalia (Fig. 2D) with gonocoxite elongate, cylindrical, almost straight; gonostylus as long as gonocoxite, slightly bent, tapering distally, apically bifid, tip seemingly broken into several parts in the holotype but complete in other paratype specimens in the same piece of amber and thus reconstructed in Fig. 2D. Epandrium rectangular, surstyli elongate, boot-shaped, distally with two short strong claw-like tenacula. Aedeagal guide (Fig. 2E) visible in lateral view, bifid; seemingly each side with a distal prolongation pointing towards tergite 9; beneath the aedeagus a digitate structure arises (probably distiphallus) whose origin is not detectable. Alongside the aedeagal guide are paired, blade shaped structures, probably parameres.

*Remarks*: The two samples SMF Be 2379 and SMF Be 2376 each contain several males of *Datzia bispina* but no females. Inverted terminalia with torsion only by segment 9 with a basal ring occurs only in extant Psychodinae. The shape of male mouthparts may indicate that at least females most likely possess funtional mouthparts.

*Datzia setosa* **n. sp.**

urn:lsid:zoobank.org:act:5C8AEAFA-607B-491A-A31C-3B906B57111F.

**Figure 4:**
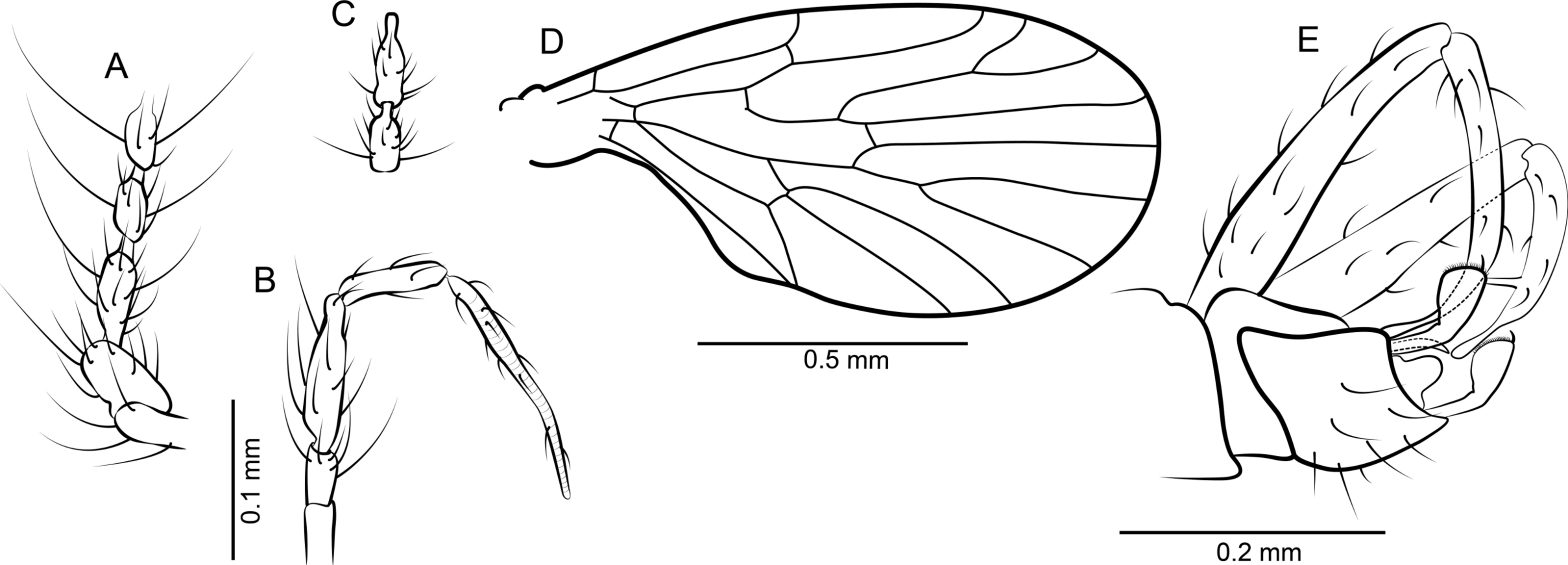
*Datzia setosa* n. sp. ♂. (A) Scape, pedicel and flagellomeres 1–3. (B) Palpus. (C) Terminal two flagellomeres. (D) Wing. (E) Terminalia, ventro-lateral view.

Figs. 4A–4E, 7B and 7D

*Material*: Holotype male SMF Be 2380.1. Syninclusions: Colembolla (>10); Acari (3); Heteroptera damaged (1); Coleoptera, Staphylinidae without head (1); Diptera, Psychodidae incomplete (1); Tanaidacea (1).

*Type-locality*: Hukawng Valley of the northern state of Kachin.

*Stratigraphic horizon*: 98.79 ± 0.62 million years, Upper Cretaceous (earliest Cenomanian) (Shi et al., 2012).

*Derivation of name:* Refers to the brush-like setose surstyli.

*Diagnosis*: A small psychodid with spare pilosity, surstyli sclerotized, elongate, with many short setae along distal margin and one claw-like tenaculum each.

*Description*: Body length: 1.66 mm.

*Head*: Without eye bridge, oval eyes covering a great part of the head. Antenna (Figs. 4A and 4C) with scape elongate, pedicel wider; flagellomeres asymmetrically articulated with a short but distinct internode which is about 1/3 the length of the flagellomere. Flagellomere 1 longer than subsequent elements, flagellomere 1 and flagellomere 2 in a ratio of: 1.57. Ascoids not distinguishable from hairs. Relative proportions of antennomeres: 10-9-11-7-7-7-7-7-7-6-6-6-7-7-7-9; apical flagellomere with apiculus. Mouthparts elongate with long thin blade-shaped mandibles, as for biting (even in males). Maxilla and labrum also elongate. Palpus (Fig. 4B) five segmented although division between segment 1 and 2 hardly visible, relative proportions of palpomeres: 10-10-25-23-40, terminal segment soft and with secondary annulations.

*Thorax*: Mesonotum setose, not visible in dorsal view but with 2 rows with apparently more than 6 dorsocentral bristles each. Tibia with long bristles, tarsal segments cylindrical, longer than wide.

*Wing*: (Fig. 4D) Broad, scarcely setose, setae along wing veins. Sc elongate, ending in costa, at approximately 90° distally to the origin of Rs, with short sc-r cross-vein. Humeral vein present, before level of radial origin. Radius with 5 veins, stem of radial fork slightly longer than R_2_. R_5_ ending at wing tip, with basal cross-vein towards stem of M_1+2_. Basal part of Rs with a short peduncle into cell br. Media with 3 veins; M_3_ and CuA_1_ originate at close quarters near the tip of cell bm. Anal vein exceptionally long, straight. CuA_2_ and A veins converge distally and terminate close to one another at wing margin. Posterior margin of wing at base slightly convex, so the anal lobe is accentuated. Cells br and bm closed. Wing length 1.16 mm.

*Abdomen*: Terminalia (Fig. 4E) visible in ventro-lateral aspect only. Gonocoxite thinner than in *T. bispina*, about 5 times longer than wide, cylindrical. Gonostylus about as long as gonocoxite, ventally curved and abruptly decreasing in width at distal fourth, thus with a slender thumb-like distal portion, and with one long spine arising from the inner surface on distal half of which only the basal portion is visible. Epandrium rectangular, apically with tergum 10 ending in a broad rounded lobe between surstyli. Surstylus slightly angulate, with a slim cylindrical basal trunk and distally cup-shaped with one strong claw-like seta and numerous short setae along apical margin. Above the epandrium there is a spine-like structure that could be one end of the penis rods or part of the aedeagal sheath, so aedeagal complex and parameres not described.

*Datzia* sp.

Figs. 5A–5E and 7E

**Figure 5:**
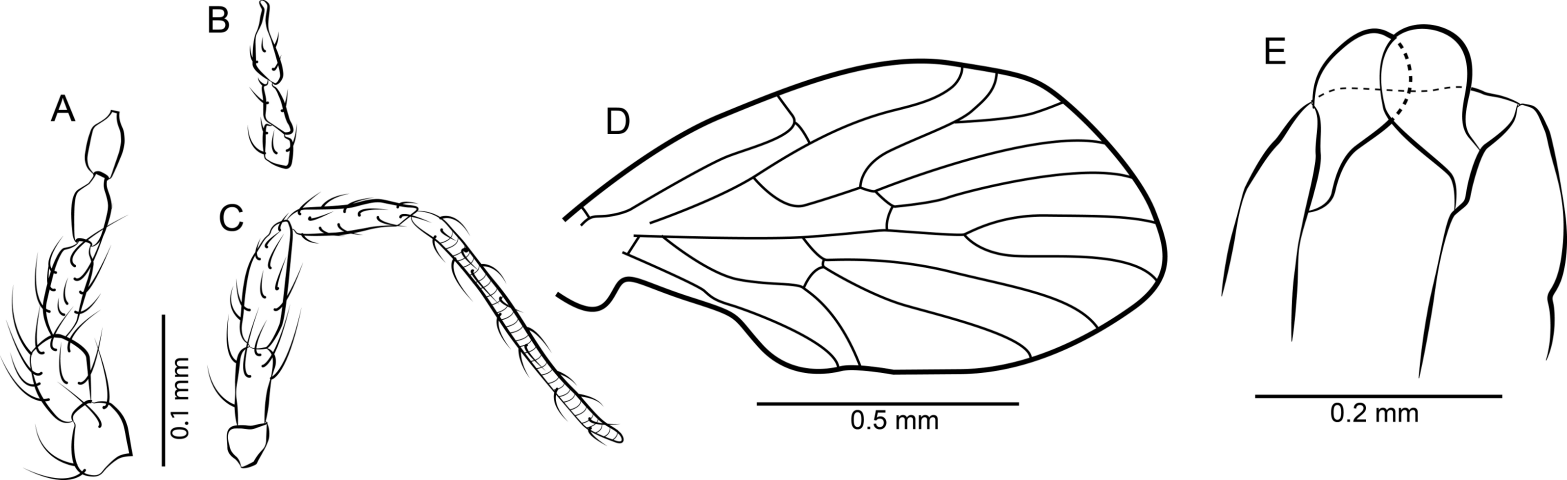
*Datzia* sp. ♀. (A) Scape, pedicel and flagellomeres 1–3. (B) Terminal 2 flagellomeres. (C) Palpus. (D) Wing. (E) Terminalia ventral view.

*Material*: Female SMF Be 2381.1. Syninclusions: Diptera, Phoridae (1); legs of two different insects.

*Locality*: Hukawng Valley of the northern state of Kachin.

*Stratigraphic horizon*: 98.79 ± 0.62 million years, Upper Cretaceous (earliest Cenomanian) (Shi et al., 2012).

*Description*: Body length without head: 1.1 mm.

*Head*: Eyes rounded without eye bridge. Antenna (Figs. 5A and 5B) with 16 antennomeres, scape short, pedicel oval, flagellomeres bottle-shaped, with nodes cylindrical and slightly eccentric and short internodes, distal segments appear asymmetrically jointed with angular basal margin, terminal flagellomere with apiculus. Relative proportions of antennomeres (length of antennomeres 8–14 is not given since articles are not arranged in a single plane): 10-11-12-10-8-8-8-. Ascoids not visible. Mouthparts elongate, possibly functional. Palpus (Fig. 5C) 5-segmented, palpal segment 5 soft and striated, about as long as segments 2 + 3 + 4; relative proportions of palpomeres: 10-16-22-26-64.

*Thorax*: Broad, similar to *Datzia bispina*.

*Left Wing*: (Fig. 5D) Distorted due to embedding process, Sc elongate, ending in costa distal to the origin of Rs, with short sc-r cross-vein, humeral vein present. Radius with 5 veins, vein R_2+3_ longer than veins R_2_ and R_3_. R_5_ ending at about wing tip (visible in right wing only since left one is distorted). Anal vein long, with a median discontinuity and reaching the wing margin near the tip of CuA_2_. Cells br and bm closed. Anal lobe accentuated. Wing length: about 1.5 mm.

*Abdomen*: With eight clearly visible segments. Tip of abdomen blunt, cerci (Fig. 5E) racket-shaped with a short bent basal stem.

*Remarks*: This female clearly belongs to the new genus *Datzia* by the wing venation and general body characteristics. However, it is not possible to relate it with or separate it from any of the previously described males and for this reason it is described but not named.

*Mandalayia***n. gen**.

urn:lsid:zoobank.org:act:7190BF4B-65D9-46DD-8707-0817D481E6F6.

*Type-species*: *Mandalayia beumersorum*

*Derivation of name: Mandalay*, the second-largest city in Myanmar.

*Diagnosis*: Very weak developed eye bridge; wing slender, lanceolate, apex rounded, without accentuated anal lobe, R_5_ originates distal to R_2+3_ + R_4_ fork, CuA_2_ and A_1_ terminate in costa at some distance; male surstyli without setae or tenacula.

*Remarks*: *Mandalayia* differs from the genus *Datzia* in the above combination of characters and is therefore classified as a new genus which is at present monospecific.

*Mandalayia beumersorum* **n. sp.**

urn:lsid:zoobank.org:act:575A0DF6-8F3C-434B-8264-67C87874727D.

**Figure 6:**
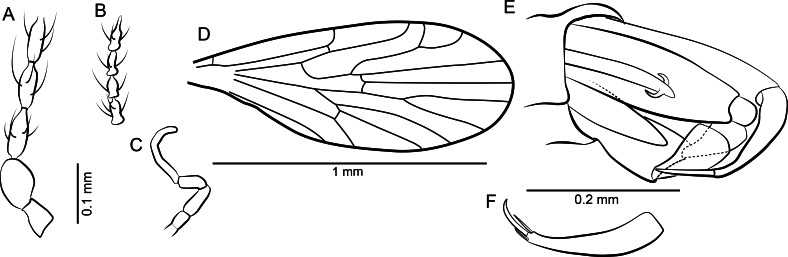
*Mandalayia beumersorum* n. sp. ♂. (A) Scape, pedicel and flagellomeres 1–3. (B) Terminal 4 flagellomeres. (C) Palpus. (D) Wing. (E) Terminalia lateral view. (F) Gonostylus.

**Figure 7:**
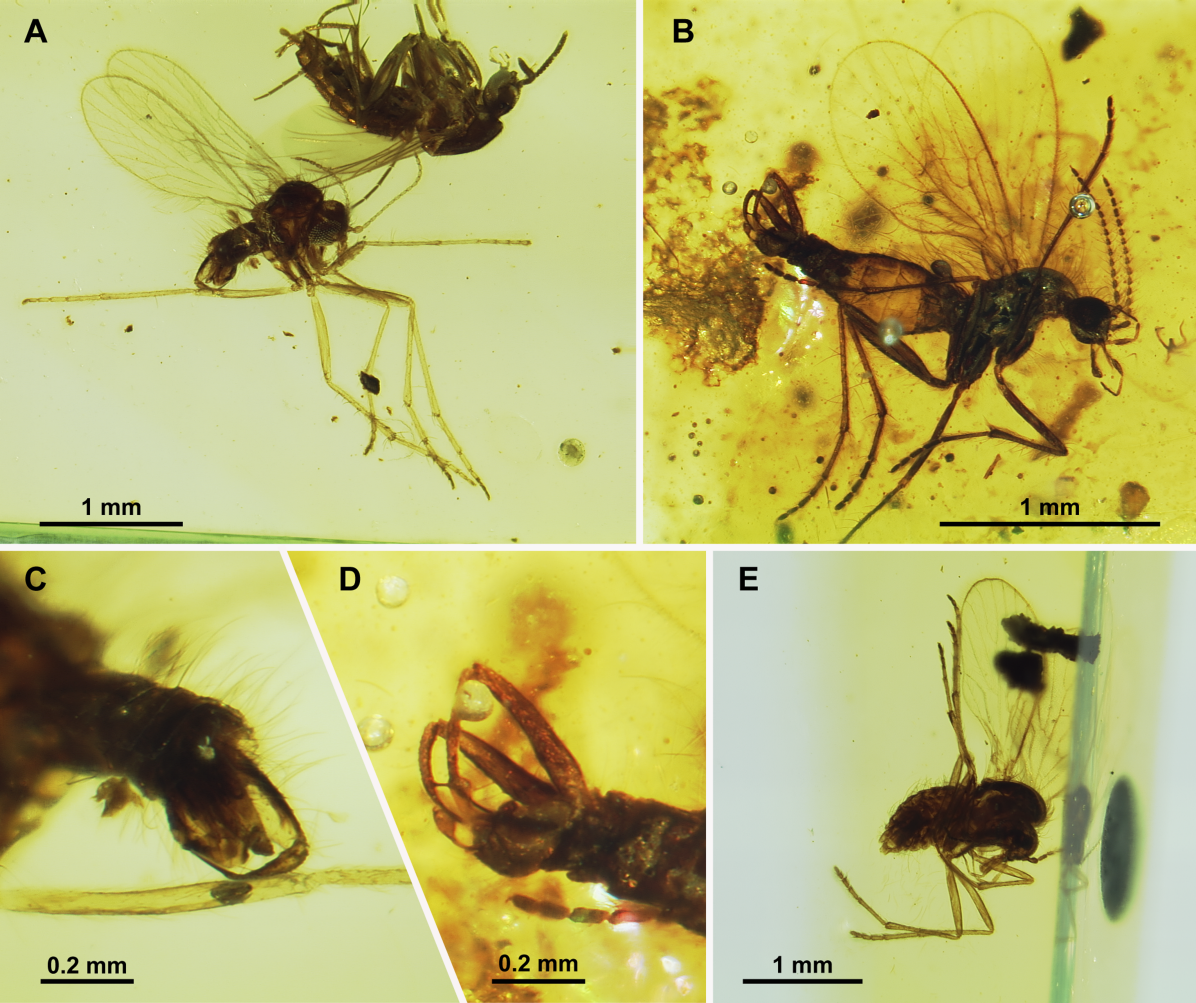
Protopsychodinae n. subfam. (A) *Mandalayia beumersorum* n. sp. ♂, Holotype specimen. (B) *Datzia setosa* n. sp. ♂, Holotype specimen. (C) *Mandalayia beumersorum* n. sp. ♂, Holotype: terminalia lateral view. (D) *Datzia setosa* n. sp. ♂, Holotype: terminalia ventro-lateral view. (E) *Datzia* sp. ♀.

Figs. 6A–6F, 7A and 7C

*Material*: Holotype male SMF Be 2385.2. Syninclusions: Psychodidae, *Phlebotomites aphoe* n. sp. (SMF Be 2385.1); Diptera, Scatopsidae (1).

*Type-locality*: Hukawng Valley of the northern state of Kachin.

*Stratigraphic horizon*: 98.79 ± 0.62 million years, Upper Cretaceous (earliest Cenomanian) (Shi et al., 2012).

*Derivation of name:* Dedicated to Mareike and Hans-Josef Beumers.

*Diagnosis*: Gonostylus with three spiniform setae at apex, the median apical one very strong, the preapical lateral two smaller, aedeagal guide apparently not bifid.

*Description*: Body length without head: 1.0 mm.

*Head:* Eyes oval, with very weakly developed eye bridge extending about 1–2 facet rows above base of scape. Antenna (Figs. 6A and 6B) with 14 flagellomeres, scape short pipe shaped, pedicel oval. Flagellomeres more-or-less flask-shaped with very short internode, apical flagellomere longer than the preceding ones with long apiculus, distal segments slightly waisted. Relative proportions of antennomeres: 10-13-14-11-11-11-11-11-11-11-11-11-11-11-11-16; ascoids unrecognizable. Mouthparts elongate; palpus (Fig. 6C) with 5 segments, separation between palpal segment 1 and 2 only vaguely discernable, segment 2 slightly longer than segment 1, segments 3 and 4 subequal and longer than the basal segments, segment 5 flexible and longer than segments 3 + 4.

*Thorax*: mesonotum covered with long hairs, with 2 rows of about 5 dorsocentral bristles each. Legs without strong bristles.

*Wing*: (Fig. 6D) Slender, Sc ending in costa, with sc-r cross-vein. Humeral vein faint but present. Radius with 5 veins, stem of radial fork 1.5 times longer than R_3_. R_5_ ending in wing tip, R_5_ originates apical to the R_2+3_ + R_4_ fork, with cross-vein towards the stem of M_1+2_. M_3_ and CuA_1_ originate near tip of the cell bm. Anal vein long and straight; hind wing basis slightly convex, but not forming an evident anal lobe. Cells br and bm closed. Wing length 1.2 mm.

*Abdomen*: With eight segments and inverted terminalia, inversion probably only by segment 9. Terminalia (Fig. 6E) with gonocoxite elongate, pipe-shaped; gonostylus (Fig. 6F) cylindrical, tapering towards apex, as long as gonocoxite with one strong terminal and two smaller preapical spiniform setae, the large one more than half as long as gonostylus. Epandrium a rectangular plate, surstyli seemingly fleshy, without tenacula, setae or spines. Between the gonocoxites lies a pipe-shaped simple structure (probably the aedeagal guide) with a pair of laterally pointed tips near apex which might represent the penis rods of the aedeagus.

Subfamily: Sycoracinae Jung, 1954

*Palaeoparasycorax* **n. gen.**

urn:lsid:zoobank.org:act:B4539700-0E5D-412B-A345-AD48E5EEBB99.

*Type- species*: *Palaeoparasycorax globosus* n.sp.

*Derivation of name*: *Palaeo*, Greek preposition (=old) and *Parasycorax*, name of extant genus.

*Diagnosis*: Antenna with 13 flagellomeres, ascoids not discernable, terminal palpus segment nearly twice as long as length of previous two segments combined, sclerotized and distally broader than at its base; wing with Sc short and terminating in acute angle in R_1_, sc-c crossvein present; number of visible abdominal segments reduced (i.e., less than 8); male terminalia inverted.

*Remarks*: *Palaeoparasycorax* differs from all remaining Sycoracinae in the shape of the terminal palpomere that is exceptionally long and seemingly slightly sclerotized and apically dilated. Furthermore, *Palaeoparasycorax* can be distinguished from the genus *Sycorax* by the inversion of male genitalia, which are of non-inverted type in *Sycorax* (Wagner & Ibáñez-Bernal, 2009; Duckhouse, 1972), and from *Parasycorax* in most characters of the wing venation (after Dos Santos, Ferreira & Bravo, 2009) as sc-c crossvein present, Rs reaching R_1_ and R_5_ complete at base.

*Palaeoparasycorax globosus* **n. sp.**

urn:lsid:zoobank.org:act:18F913A7-6A8D-4E43-924B-E41392850903.

**Figure 8:**
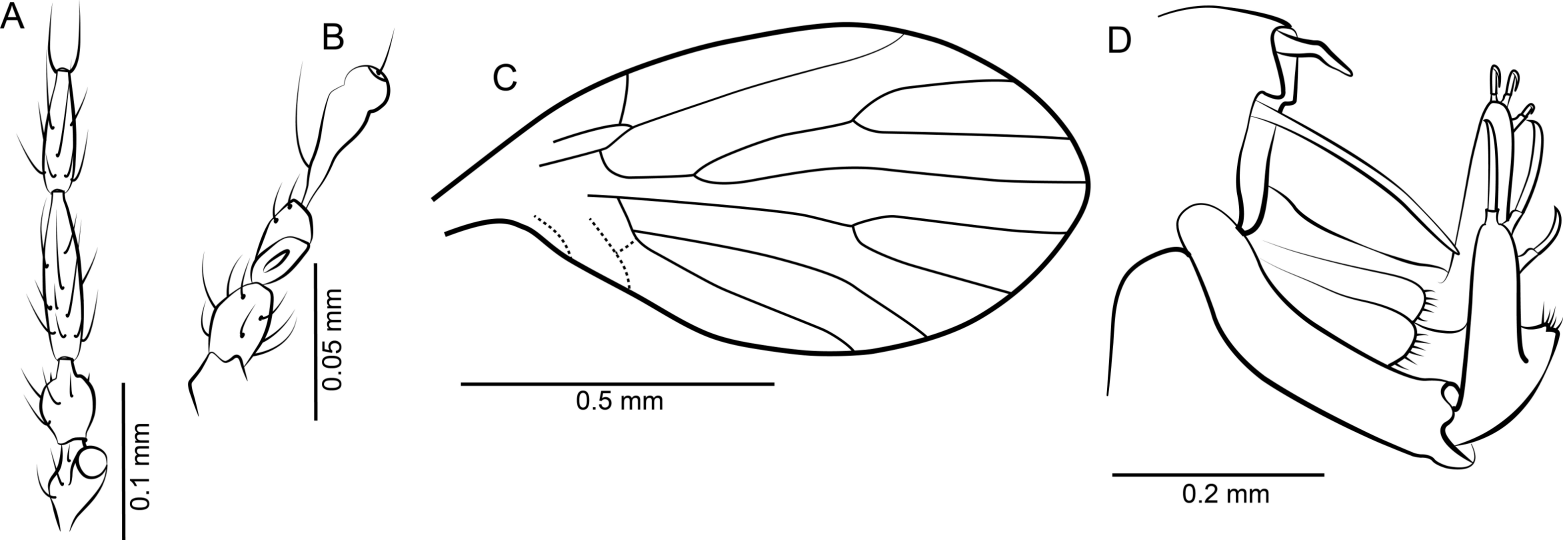
*Palaeoparasycorax globosus* n. sp. ♂. (A) Scape, pedicel and flagellomeres 1–3. (B) Palpus. (C) Wing. (D) Terminalia lateral view.

Figs. 8A–8D, 11A, 11B and 11D.

*Material*: Holotype male SMF Be 2389.1. Paratype male SMF Be 2386.1. Syninclusions SMF Be 2389: Diptera, Mycetophilidae (2); Diptera, Empididae (1); Diptera, Chironomidae (4); Psocoptera (1); Hymenoptera, Chalcidoidea (1). Syninclusions SMF Be 2386: Diptera, Scatopsidae (1); Diptera, Brachycera (1).

*Type-locality*: Hukawng Valley of the northern state of Kachin.

*Stratigraphic horizon*: 98.79 ± 0.62 million years, Upper Cretaceous (earliest Cenomanian) (Shi et al., 2012).

*Derivation of name*: Refers to the globular ending of the terminal segment of palpus.

*Diagnosis:* Scape with a lateral cavity; palpomere 3 with an elongate slit; male terminalia with gonostyli having a small inner protuberance and 3 strong terminal and subterminal spiniform setae.

*Description*: Body length without head: 1.05 mm.

*Head:* with oval eyes. Antenna (Fig. 8A) longer than wing, with 13 flagellomeres. Scape short, with a lateral prologation with a circular aperture, pedicel subspherical, with a short but evident apical neck. Flagellomeres elongate, articulated symmetrically, ascoids unrecognizable. Flagellomere 1 longest, following flagellomeres decreasing in length and diameter towards terminal one, terminal segment with slender apiculus which is about half as long as segment. Relative proportions of antennomeres: 10-11-20-16-14-13-14-13-11-11-11-11-11-11-11. Mouthparts short, non-functional, palpus (Fig. 8B) 4 segmented, segment 3 with an oblique slit, terminal segment slightly sclerotized increasing in width distally and ending in a globular structure with a distal seta. Palpus segment 1 basally not visible, relative proportions of terminal 3 palpomeres: ?-7-7-13.

*Thorax*: Mesonotum setose. Legs elongate without distinctive features.

*Wing*: (Fig. 8C) Length 1.15 mm, oval, with R_4+5_ ending at the broadly wing apex. Sc short, ending in a sharp angle in Radial vein, with a long and faint sc-c crossvein. Radius with 4 veins, apparently R_4+5_ fused. Radial and medial forks at about the same level in the distal third of the wing. Anal vein short.

*Abdomen:* With six visible segments.

*Terminalia*: (Fig. 8D) Inverted, epandrium seemingly rectangular, cerci oval. Gonocoxites are displaced beside epandrium, elongate pipe shaped, slightly bent. Gonostyli upright, complex, basally with a small inner protuberance with some terminal small setae. Distal portion blunt with one terminal and two subterminal long and strong spiniform setae, each originates from a basal tubercle. Aedeagus seemingly a thin elongate straight sclerite. Another shorter median lobe located ventral to the aedeagus is present (part of the parameres?), but cannot be named without doubt.

*Palaeoparasycorax suppus* **n.sp.**

urn:lsid:zoobank.org:act:6CB43B4E-83D5-42B3-ACE4-0D809C79FA08.

**Figure 9:**
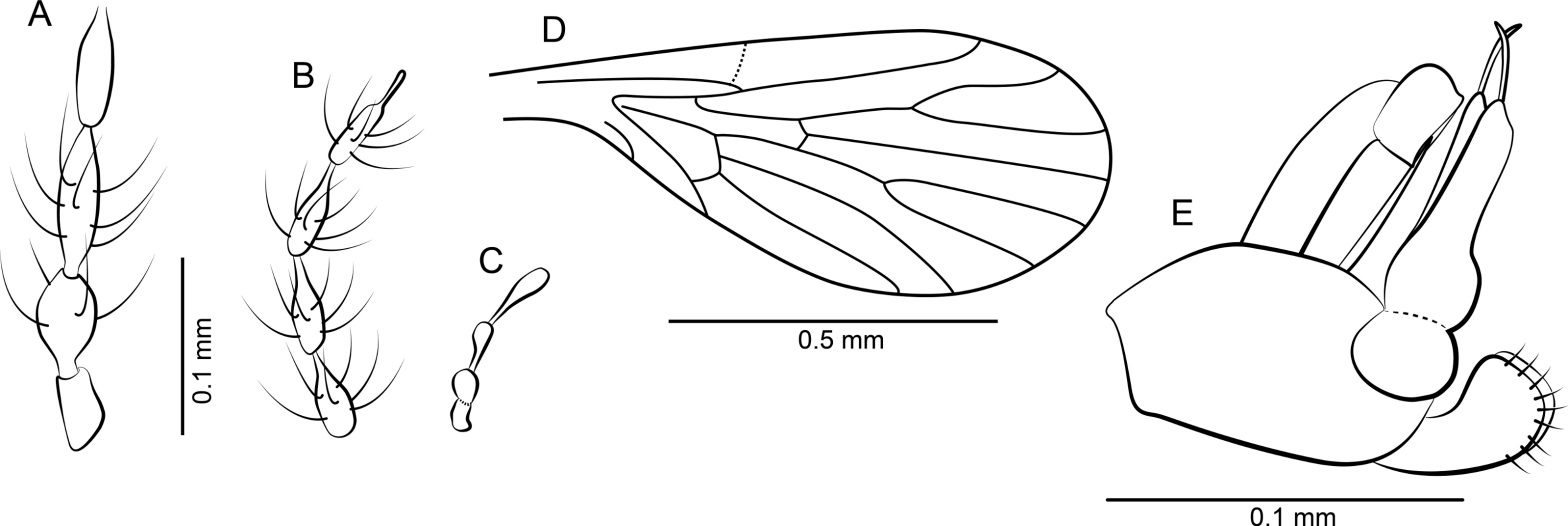
*Palaeoparasycorax suppus* n. sp. ♂. (A) Scape, pedicel and flagellomeres 1–2. (B) Terminal 4 flagellomeres. (C) Palpus. (D) Wing. (E) Terminalia lateral view.

Figs. 9A–9E and 18E.

*Material*: Holotype male GPIH no. 4418, collection Gröhn no. 11006. Syninclusions: Psychodidae, *Parasycorax simplex* n. sp. (GPIH no. 4449, collection Gröhn no. 11020); Psychodidae, *Bamara groehni* n. sp. (GPIH no. 4417, collection Gröhn no. 11006); Psychodidae, *Phlebotomus* sp. (collection Gröhn no. 11019); Psychodidae, Phlebotominae sp. (collection Gröhn no. 11020).

*Type-locality*: Hukawng Valley of the northern state of Kachin.

*Stratigraphic horizon*: 98.79 ± 0.62 million years, Upper Cretaceous (earliest Cenomanian) (Shi et al., 2012).

*Derivation of name: suppus*, Latin (=upright), because of the upright gonostyli and aedeagus.

*Diagnosis*: Male terminalia with gonocoxites and gonostyli in a dorsal position besides epandrium, gonostyli each with one strong apical spine, aedeagus upright directed.

*Description*: Body length: 0.65 mm.

*Head:* About circular, eyes oval, no eye bridge. Antenna (Figs. 9A and 9B) approximately 1.74 mm long, with 13 flagellomeres. Scape pipe shaped, pedicel spherical. Flagellomeres elongate bottle-shaped, necks of distal flagellomeres longest, ascoids unrecognizable. Segments slightly decreasing in length, terminal segment longer than the penultimate with apiculus which is not longer than the node. Approximate relative proportions of antennomeres: 10-6-16-14-13-13-13-12-11-11-11-11-11-10-13. Mouthparts not clearly visible, short; palpus (Fig. 9C) 4 segmented, first and second segments about equal in length; relative proportions: 10-10-13-20; apical three palpomeres rounded and broader distally.

*Thorax*: Mesonotum with long hairs. Legs long.

*Wing*: (Fig. 9D) Length approximately 0.81 mm, width 0.4 mm, hairs on veins and along wing margin. Sc short, ending in radius in acute angle, sc-c cross-vein present but hardly visible. Radius with 4 veins; R_1_ long, ending distad to the radial fork, stem of R_2+3_ behind cell br, as long as R_2_, R_3_ longer than R_2_. R_4+5_ originating near middle of wing, and about middle of M_1+2_; Radial and medial forks in the distal half of wing, but radial fork slightly distal to medial fork; M_3_ and CuA_1_ emerging separately from the closed cell bm; CuA_2_ short, ending at wing margin before the origin of CuA_1_; anal vein short.

*Abdomen:* With six visible segments, distal segments completely hidden in segment 6. Segment 1 small, segment 3 the largest.

*Terminalia*: (Fig. 9E) Inverted (probably by the hidden segments 7 and 8). Gonocoxites and gonostyli in a dorsal position beside epandrium, epandrium not visible. Gonocoxite large and broad, gonostylus upright, 1.5 times longer than gonocoxite, basally wider, tapering distally, with a strongly sclerotized apical spine that is about 1/3 of the total length of the gonostylus. Between the gonocoxites is the upright pipe-shaped aedeagus that has a slighter sclerotized tip. Cerci with a broad rounded apex, setose, fleshy. The thin spine, if not an artifact, maybe a paramere.

*Remarks*: the specimen can be assigned to *Palaeoparasycorax* based on shape and sclerotization of the terminal palpus segment, wing with Sc short and terminating in acute angle in R_1_, sc-c crossvein present, only 6 abdominal segments visible and male terminalia inverted. It can be distinguished from *Palaeoparasycorax globosus* by the absence of a lateral cavity on the scape, palpomere 3 without an elongate slit and the shape of gonocoxite, gonostylus and number of spines on gonostylus.

Genus: *Parasycorax* Duckhouse, 1972.

*Parasycorax simplex* **n. sp.**

urn:lsid:zoobank.org:act:43D74B77-031E-435F-9FF9-AA25C1BFBEC1.

**Figure 10:**
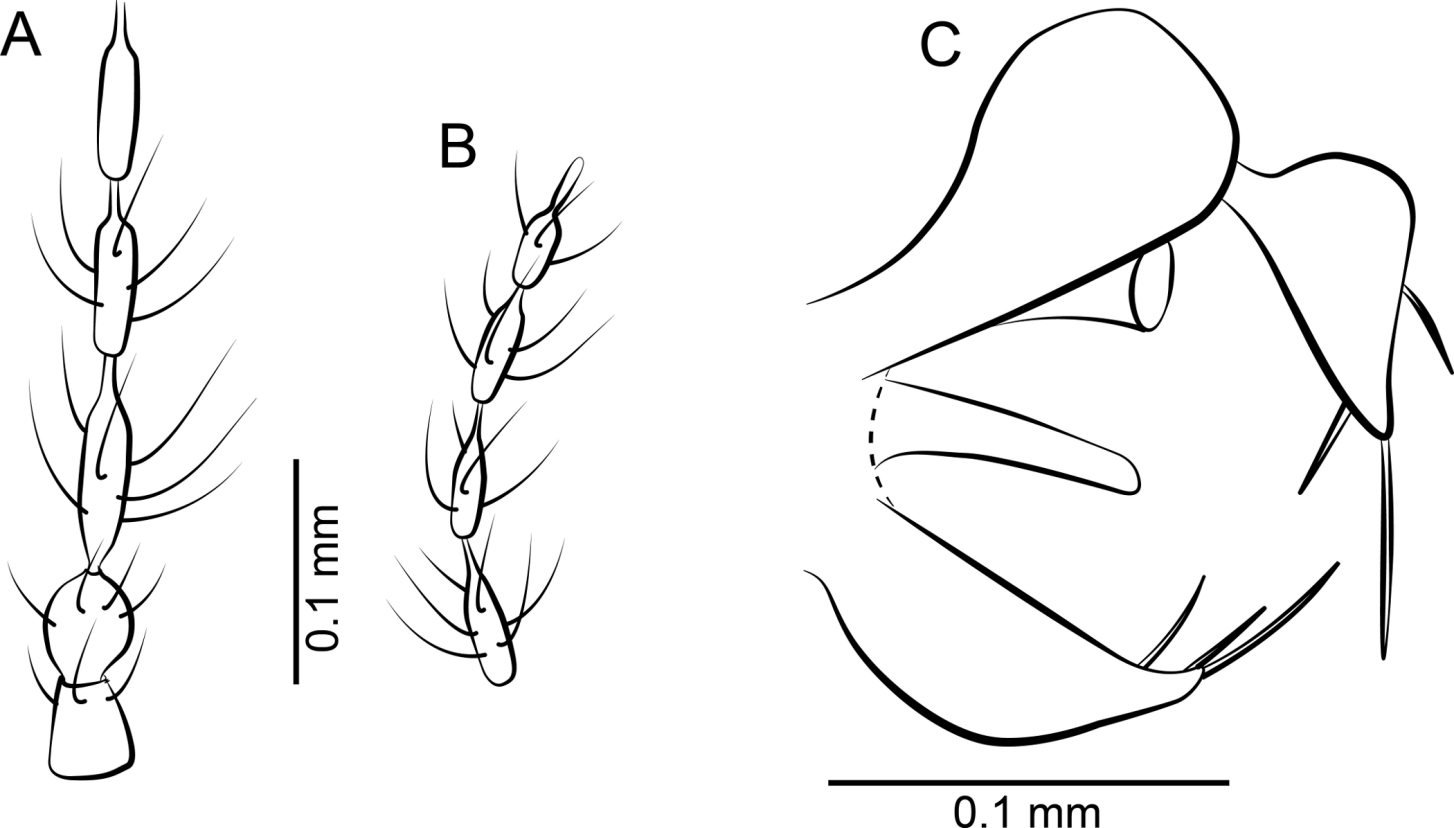
*Parasycorax simplex* n. sp. ♂. (A) Scape, pedicel and flagellomeres 1–3. (B) Terminal 4 flagellomeres. (C) Terminalia lateral view.

**Figure 11:**
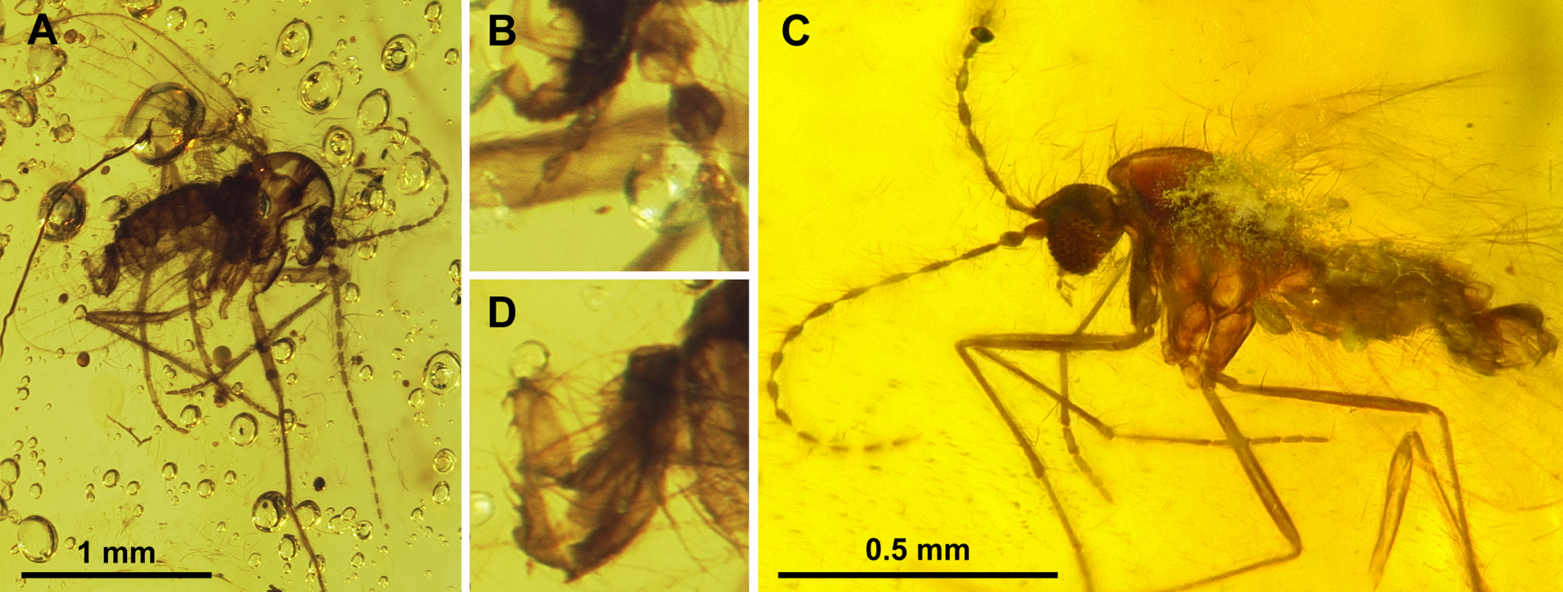
Sycoracinae. (A) *Palaeoparasycorax globosus* n. sp. ♂, Holotype specimen. (B) *Palaeoparasycorax globosus* n. sp. ♂, Holotype: detail of antenna and palpus. (C) *Parasycorax simplex* n. sp. ♂, Holotype specimen. (D) *Palaeoparasycorax globosus* n. sp. ♂, Holotype: terminalia lateral view.

Figs. 10A–10C, and 11C.

*Material*: Holotype male GPIH no. 4449, collection Gröhn no. 11020. Syninclusions: Psychodidae, *Bamara groehni* n. sp. (GPIH no. 4417, collection Gröhn no. 11006); Psychodidae, *Palaeoparasycorax suppus* n. sp. (GPIH no. 4418, collection Gröhn no. 11006); Psychodidae, *Phlebotomus* sp. (collection Gröhn no. 11019); Psychodidae, Phlebotominae sp. (collection Gröhn no. 11020).

*Type-locality*: Hukawng Valley of the northern state of Kachin.

*Stratigraphic horizon*: 98.79 ± 0.62 million years, Upper Cretaceous (earliest Cenomanian) (Shi et al., 2012).

*Derivation of name*: *Simplex*, Latin (=uncomplicated), because of the simplicity of the male terminalia.

*Diagnosis*: terminal flagellomere with long apiculus; gonocoxites stout and laterally convex, gonostylus with one short middle seta, one preapical seta, and an elongate distal seta.

*Description*: Body length: 0.9 mm.

*Head:* Wider than long, eyes oval, no eye bridge. Antenna (Figs. 10A and 10B) approximately 1 mm long, with 13 flagellomeres. Scape short, pedicel spherical. Flagellomeres elongate, hairy, bottle-shaped with short internode, articulated symmetrically; ascoids unrecognizable. Antennomeres slightly decreasing in length, terminal flagellomere longer than penultimate, with long apiculus. Approximate relative proportions of antennomeres: 10-12-24-22-20-20-20-20-20-18-18-18-18-14-18. Mouthparts short, palpus with 3 visible segments: first and second segment about equal in length, oval; segment 3 thinner and slightly longer than previous two combined.

*Thorax*: Mesonotum setose, seemingly with 2 rows of dorsocentral bristles. Legs elongate. Tibia 1 with several long hairs on inner side and apically; first tarsomere as long as tarsomeres 2-5.

*Wing*: Length approx. 0.75 mm. Veins not sufficiently visible to describe. Radius with 4 veins.

*Terminalia*: (Fig. 10C) Inverted, damaged so that shape of individual parts is difficult to judge. Epandrium and surstyli unrecognizable. Gonocoxites stout strong, laterally convex. Gonostylus appears elongate triangular in lateral view, tapering distally, with an inner and a lateral short spiniform, and an elongate distal seta. The apical spiniform seta is about as long the gonostylus. Between the gonocoxites lies a digitate aedeagus.

*Remarks*: the species resembles extant representatives of *Parasycorax* in the inversion of male terminalia, the gereral shape of the gonostylus and in having more than one spine on the gonostylus. It differs from *P. bidentis* in the shape of the gonostylus, which is without basal sclerotized arms in *P. simplex*, and from *P. bidentis*, *P. filipinae* and *P. uritriensis* in having three spines on the gonostylus instead of two in the former two and 15 in the latter species. *P. simplex* can be distinguished from *P. satchelli* in the arrangement of setae on the gonostylus, the shape and size of the gonocoxite and in having the terminal flagellomere with a long apiculus.

Subfamily: Phlebotominae Rondani, 1840.

Tribe: Hertigiini Abonnenc and Leger, 1976.

Subtribe: Idiophlebotomina Artemiev, 1991.

Genus: *Phlebotomites* Hennig, 1972.

*Phlebotomites*Hennig, 1972, 39. Type: *Phlebotomites brevifilis* Hennig (original designation).

*Diagnosis*: Eyes without eye bridge; mouthparts well developed; first flagellomere long; last palpomere shorter or equal to the preceding one; wing with a broad distal half and broadly rounded tip; Rs four branched; R_2_ and R_3_ separated; origin of R_4_ apical to origin of R_5_; male terminalia phlebotomine-like (Hennig, 1972; Lewis, 1982).

*Previously known species*: *Phlebotomites brevifilis*Hennig, 1972, *Phlebotomites longifilis*Hennig, 1972, *Phlebotomites burmaticus*Ain Malak, Salamé & Azar, 2013, *Phlebotomites grimaldii*Ain Malak, Salamé & Azar, 2013, and *Phlebotomites neli*Ain Malak, Salamé & Azar, 2013.

*Phlebotomites aphoe* **n. sp.**

urn:lsid:zoobank.org:act:BFE03C00-720A-4186-BD34-646D9B8BF7F4.

**Figure 12:**
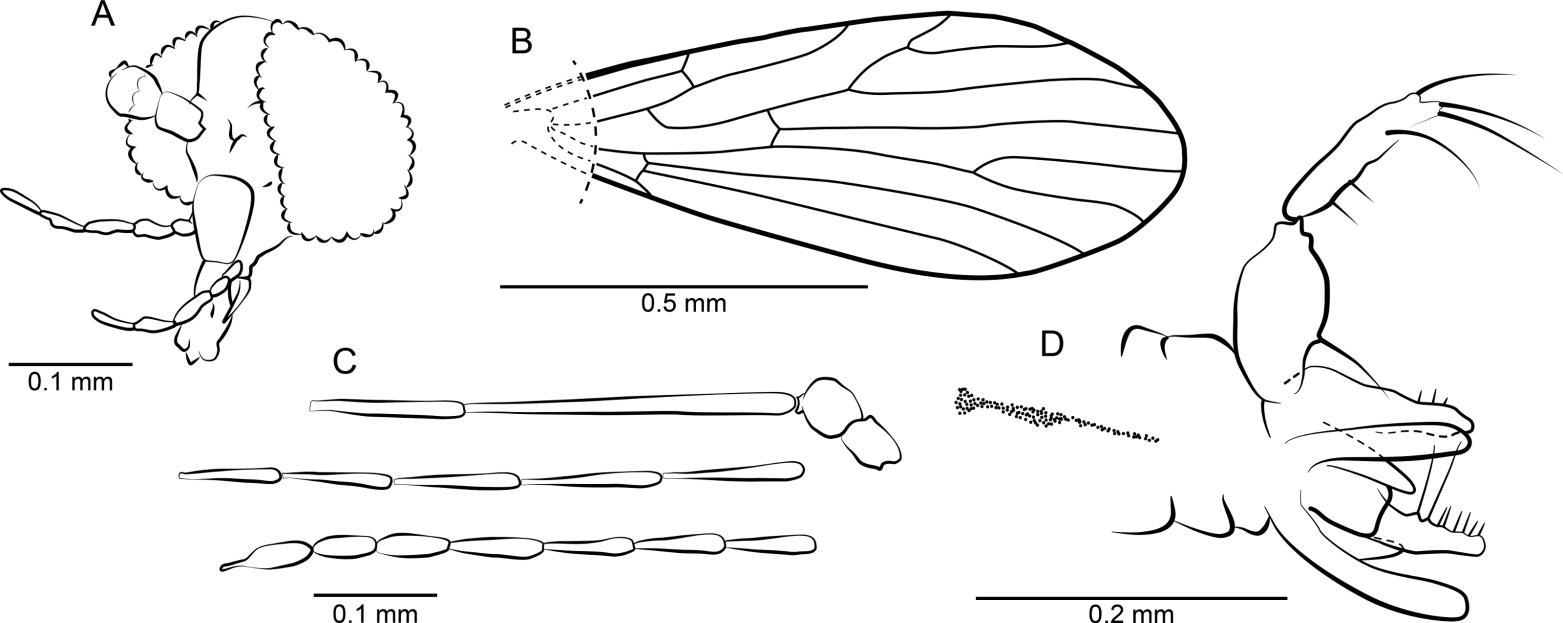
*Phlebotomites aphoe* n. sp. ♂. (A) Head. (B) Wing. (C) Antenna. (D) Terminalia ventral view.

Figs. 12A–12D and 14C.

*Material:* Holotype male SMF Be 2385.1. Syninclusions: Psychodidae, *Mandalayia beumersorum* n. sp. (SMF Be 2385.2); Diptera, Scatopsidae (1).

*Type-locality*: Hukawng Valley of the northern state of Kachin.

*Stratigraphic horizon*: 98.79 ± 0.62 million years, Upper Cretaceous (earliest Cenomanian) (Shi et al., 2012).

*Derivation of name:* From Burmese language, *a phoe*, grandfather.

*Diagnosis*: A small phlebotomine species characterized by the wing branching pattern, as compared with other described species of *Phlebotomites*.

*Description*: Body length 1.00 mm.

*Head*: (Fig. 12A) Length 0.21 mm, width 0.25 mm, slightly wider than long. Eyes rounded, without eye bridge above antennal insertions; frons with two short separate sutures at level of the eye angle above antennal insertions. Clypeus longer than wide, broader in the upper margin. Proboscis relatively long, 0.10 mm, not longer than one-half the length of head; labrum just surpassing the level of distal margin of labella; labella compact; maxilla lacinia blade-like, clear; palpus with 5 segments, apical segment 5 as long as segment 4; palpus length: 0.175 mm, formula: 10-17-20-20-20. Antenna (Fig. 12C) length 1.415 mm; antennal scape cylindrical, pedicel spherical and flagellum composed by 14 symmetric flask-shaped flagellomeres, except last three which are cylindrical; first flagellomere very long, nearly as long as head and proboscis, and 1.5 times as long as palpus; proportion flagellomere 1/ flagellomere 2: 2.14, and nearly with the same length than flagellomere 2 + 3; terminal flagellomere with long apiculus which is as long as 0.5 the length of its body; ascoids not seen.

*Thorax*: Phlebotomine-like, legs lost.

*Wing*: (Fig. 12B) Length: 0.95 mm, maximal width: 0.35 mm, apically rounded, R_5_ ending before wing apex; veins similar in number and distribution as in other species of *Phlebotomites*, Sc double-ended, one reaching C and the other ending in R_1_; R_1_ ending at level of bifurcation R_2+3+4_; R_2_ shorter than R_2+3_ and about 0.33 the length of R_3_; R_5_ nearly two times as long as R_3_; vein r-m strong; medial fork apical to radial fork; M_3_ ending at the same level as R_3_, and CuA_1_ distal to level of median fork; CuA_2_ short but ending distal to the level of the origin of CuA_1_.

*Abdomen*: Vestiture lost. Terminalia (Fig. 12D) phlebotomine-like; gonopod length 0.23 mm, longer than epandrial lobes; gonocoxite length 0.11 mm, oval, shorter than paramere (length 0.14 mm) and gonostylus (length 0.12 mm), with one internal long basal seta; gonostylus cylindrical with four spiniform setae of which two are apical, one preapical near the apex and one at the apical 0.33, and with two perennial simple setae on basal third. Parameres simple, apex slightly curved, with two setae in the ventral margin and two long setae near the middle followed by some apical dorsal setae. Epandrial lobes 0.14 mm, as long as 5 times its maximal width, and as long as paramere, apically rounded. Cerci lobe-like reaching the level of mid portion of the epandrial lobes. Epandrium difficult to see but subquadrate ending before the apex of cerci.

*Remarks*: *Phlebotomites aphoe* n. sp. is the sixth species described of this antique genus; *Phlebotomites brevifilis* Hennig and *Phlebotomites longifilis* Hennig were described from the Lower Cretaceous of Lebanon, and *Phlebotomites burmaticus* Ain Malak, Salamé & Azar, *Phlebotomites grimaldii* Ain Malak, Salamé & Azar, *Phlebotomites neli* Ain Malak, Salamé & Azar were described from the Lower Cretaceous Burmese amber. *Phlebotomites brevifilis* differs from *Phlebotomites aphoe* by the length of R_1_ which overpasses the R_2+3+4_ fork, nearly reaching the R_2+3_ fork, and the medial fork (M_1+2_) is basal or at most at level of R_2+3_ fork, has the first flagellomere comparatively short as compared with the second flagellomere, male gonostylus very long with 5 spiniform setae, and very short epandrial lobe. *Phlebotomites longifilis* differs from *P*. *aphoe* by the length of R_1_ which just overpasses the R_2+3_ fork, and the medial fork (M_1+2_) is basal or at most at level of R_2+3_ fork, by the large gonostylus with 5 spiniform setae and the form of the paramere which is wider at middle. *Phlebotomites grimaldii* differs from *Phlebotomites aphoe* by the palpus segment proportions, having segment 5 shorter than 4, the medial fork is nearly at the same level of radial fork (R_2+3_), and male gonostylus is longer as compared with its gonocoxite that does not have a strong basal seta. *Phlebotomites neli* was described based on female specimens, but can be differentiated from *P*. *aphoe* because the medial fork is at the same level as the radial fork and the vein M_3_ originates distad to the m-cu vein. *Phlebotomites burmaticus* was described based on one female specimen, but differs from *Phlebotomites aphoe* in R_1_ reaching wing margin at level of radial and medial fork (i.e., medial fork at same level as radial fork) and that species apparently do not have the m-cu vein.

Tribe: Phlebotomini Rondani, 1840.

Subtribe: Phlebotomina Rondani, 1840.

Genus: *Phlebotomus* Rondani & Berte, 1840.

*Phlebotomus vetus* **n.sp.**

urn:lsid:zoobank.org:act:4217A01A-AB5B-47D0-845B-DFF9957390B6.

**Figure 13:**
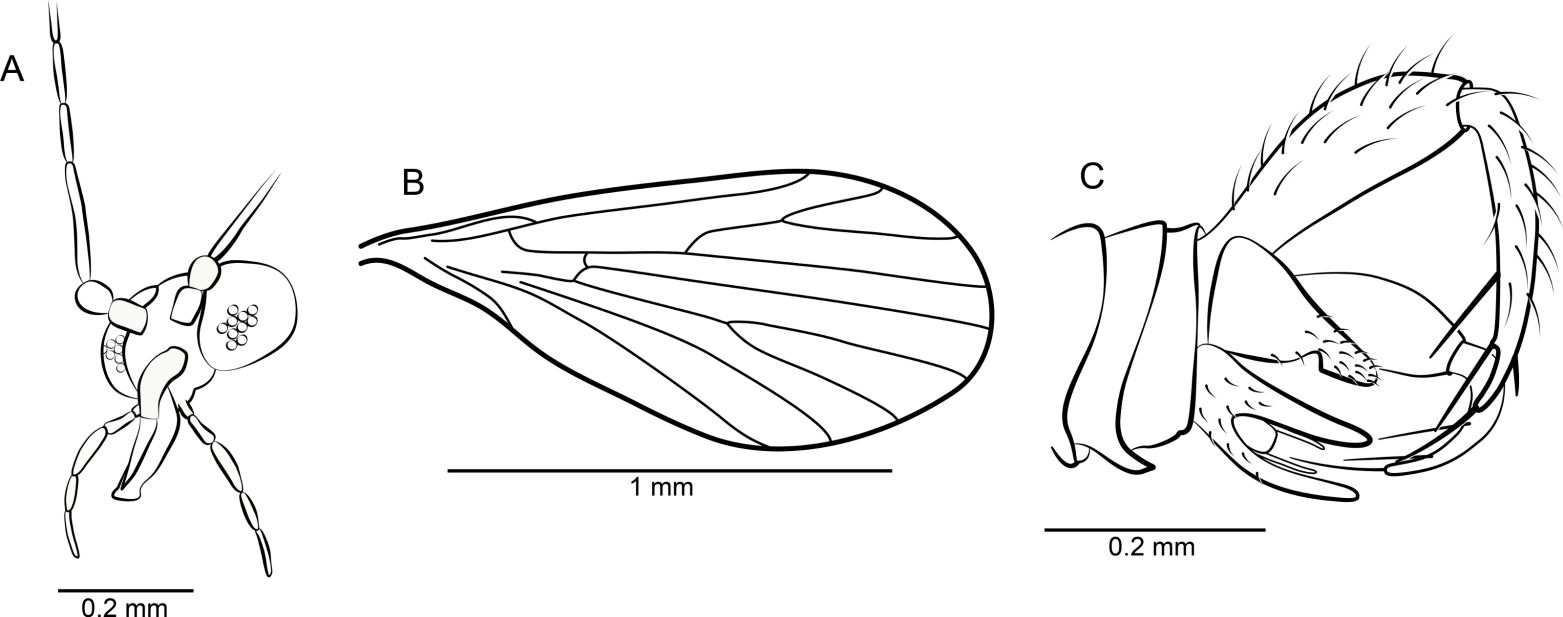
*Phlebotomus vetus* n. sp. ♂. (A) Head frontal view. (B) Wing. (C) Terminalia ventro-lateral view.

**Figure 14:**
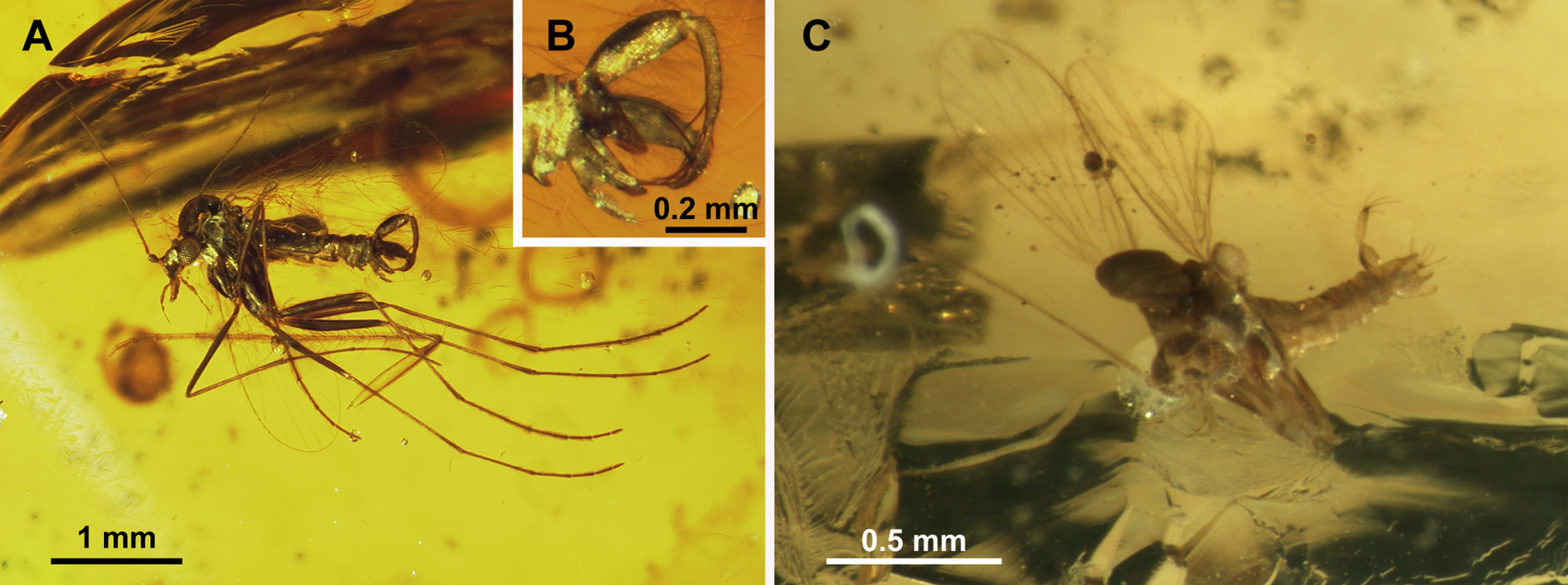
Phlebotominae. (A) *Phlebotomus vetus* n. sp. ♂, Holotype specimen. (B) *Phlebotomus vetus* n. sp. ♂, Holotype: terminalia lateral view. (C) *Phlebotomites aphoe* n. sp. ♂: Holotype specimen.

Figs. 13A–13C, 14A and 14B.

*Material*: Holotype male SMF Be 2533.

*Type-locality*: Hukawng Valley of the northern state of Kachin.

*Stratigraphic horizon*: 98.79 ± 0.62 million years, Upper Cretaceous (earliest Cenomanian) (Shi et al., 2012).

*Derivation of name: Vetus*, Latin (= old).

*Diagnosis*: Broadly rounded wing tip; gonocoxite about as long as gonostylus, gonostylus bearing 4 strong spiniform setae, paramere triangular with a dorsal preapical constriction.

*Description*: Body length without head: 1.37 mm

*Head*: (Fig. 13A) Without eye bridge, eyes oval. Antenna with 14 flagellomeres; scape short, pedicel spherical, flagellomeres elongate, ascoids not visible. Relative proportions of antennomeres: 10-14-40-18-18-18-17-18-17-17-16-16-16-14-14-14. First and second palpomere not clearly distinguishable thus relative length of first two segments is approximated: 10-9-23-14-20; terminal segment longer and thinner than penultimate but shorter than 3 + 4. Mouthparts elongate, reaching middle of palpomere 4.

*Wing*: (Fig. 13B) Length: 1.4 mm. Sc ending in R_1_, R with five branches, radial and medial forks beyond middle of wing, medial fork basad of radial fork. R_5_ ending in the broadly rounded wing tip. Tip of CuA_1_ between level of radial and medial fork, CuA_2_ short, ending before the end of Sc and about level of the origin of Rs.

*Terminalia*: (Fig. 13C) Inverted. Gonocoxite about as long as gonostylus without specialized setae. Ventral margin of gonocoxite almost straight, dorsal margin bent. Gonostylus bearing four strong spiniform setae, one inner whose base cannot be seen, and the three remaining approximately of same length and arranged as follows: one terminal seta, one preapical seta, and one seta at about the middle of length of gonocoxite. Paramere shorter than gonocoxite, triangular in general shape with a broad base tapering to rounded apex, with a dorsal preapical constriction. Epandrium with deep distal emargination so that two lateral lobes (probably epandrial lobes) develop, that are about as long as the parameres; base of lobes not clearly visible. Cerci poorly visible, probably reaching apical third of lateral lobes. Aedeagus and penis guide not visible.

*Remarks*: The species can be assigned to the subfamily Phlebotominae by elongate mouthparts even in the male, rounded eyes, palpus 5 segmented, the shape of epandrium with posterior-lateral lobes and gonostylus with several long and strong setae. Strictly, in Phlebotominae the epandrial lobes should be articulated at their base with an independent origin, not being extensions of the epandrium without articulation (as shown in Fig. 13C). However, in *Phlebotomus vetus* n. sp. the base of the epandrial lobes is only poorly visible so that the lobes appear to be fused but not articulated. The wing venation is more-or-less typical for Phlebotominae although the wing has a broader rounded tip than in extant species. The arrangement of setae on the gonostylus and the shape of the parameres with a preapical constriction distinguishes the specimen from all remaining *Phlebotomus* species.

Subfamily: Bruchomyiinae Alexander, 1920.

Genus: *Nemopalpus* Macquart, 1838.

*Nemopalpus quadrispiculatus* **n. sp.**

urn:lsid:zoobank.org:act:EB83F66F-4660-45E3-9F79-A04AF27FEAB7.

**Figure 15:**
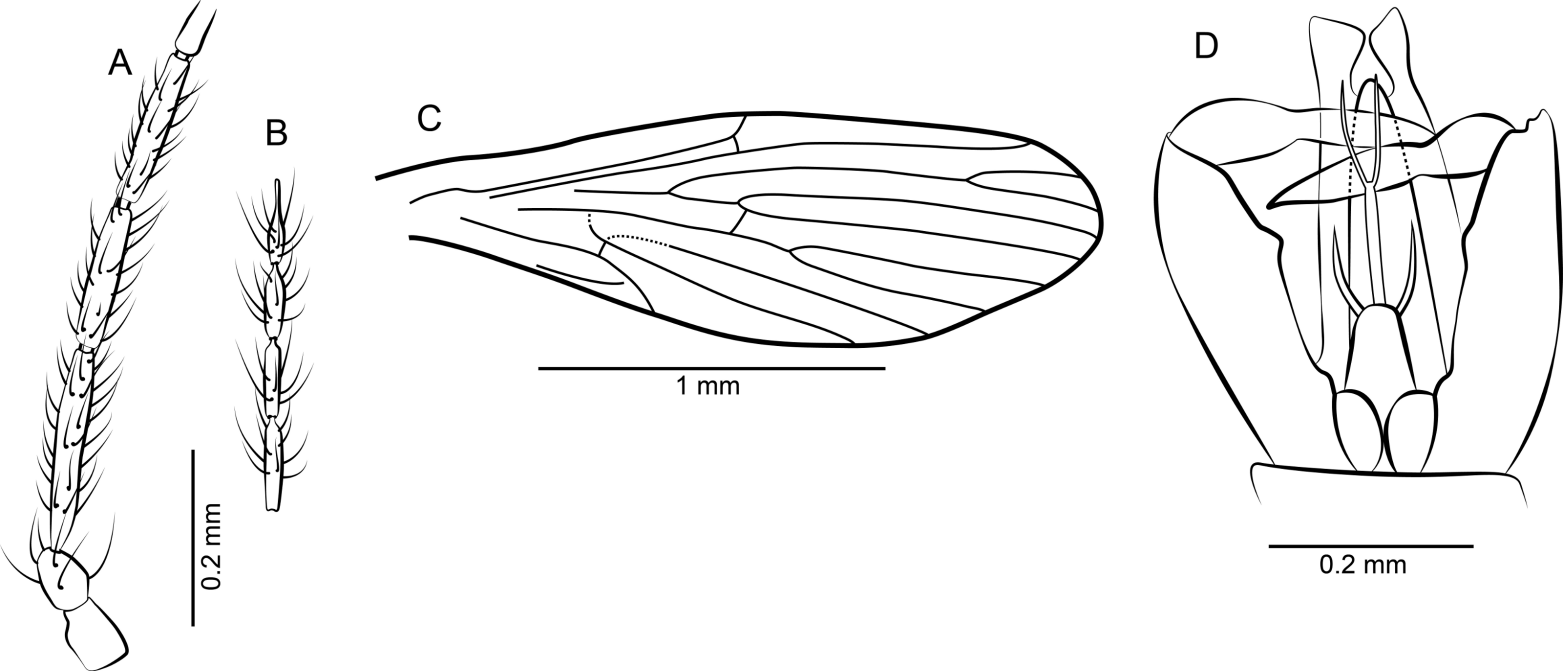
*Nemopalpus quadrispiculatus* n. sp. ♂. (A) Scape, pedicel and flagellomeres 1–4. (B) Terminal 4 flagellomeres. (C) Wing. (D) Terminalia dorsal view.

Figs. 15A–15D, 18A and 18C.

*Material:* Holotype male SMF Be 2390.

*Type-locality*: Hukawng Valley of the northern state of Kachin.

*Stratigraphic horizon*: 98.79 ± 0.62 million years, Upper Cretaceous (earliest Cenomanian) (Shi et al., 2012).

*Derivation of name*: From Latin, *quattuor* (=four) and Latin, *spiculus* (=tip), because of the 4 spine-like appendages of parameres and aedeagus.

*Diagnosis*: Small species; strongly setose; wing with radial fork rather distad; aedeagus and parameres each with a pair spine-like appendages.

*Description*: Body length: about 2.3 mm.

*Head*: Eyes roundish/oval, without eye bridge. Antenna (Figs. 15A and 15B) with cylindrical scape and oval pedicel and 14 elongate cylindrical flagellomeres with long and dense setae; terminal flagellomere with slender apiculus which is as long as the basal portion. Due to dense vestiture the presence of ascoids on flagellomeres cannot be detected. Relative proportions of antennomeres: 4-6-17-13-13-12-11-11-10-9-9-8-8-7-7-7; antennal length 2 mm. Mouthparts without functional mandibles. Palpus with four palpomeres observed, whether there is a very short 1st segment, as is usual in the subfamily, remains unknown; relative proportions of palpomeres: ?-5-18-14-27; overall length of palpus about 0.8 mm.

*Wing*: (Fig. 15C) Length: 2.1 mm, ca 3 × longer than wide; Sc ending in C at middle of wing and beyond base of R_2+3_, sc-r crossvein faint. R_1_ elongate, ending distad to R_2+3_ fork; fork R_2_ + R_3_ positioned rather distally, fork R_4_ + R_5_ at about same level as Sc tip (at middle of wing); wing tip between R_3_ and R_4_; r-m crossvein faint; fork M_1+2_ distad of fork R_4_+R_5_; M_3_ connected to M_1+2_ stem; CuA_1_ in contact at base with M_3_ and forming a closed cell bm; CuA_2_ moderately short, ending at wing margin before level of fork R_2+3_-R_4+5_. Anal vein short, almost straight, ending in wing.

*Abdomen*: With 8 pregenital segments, plus inverted terminalia, inversion by segments 8 and 9. Terminalia (Fig. 15D) with gonocoxite straight, pipe-shaped, with a small protuberance on inner margin in distal half, about 1.4 × longer than gonostylus. Gonostylus straight, distinctly waisted in the basal third; distal 2/3 long cone-shaped with acute apex. Epandrium elongate rectangular, with setose cerci articulated at base. Proctiger oval, setose. Aedeagus elongate thin, tube-shaped, with two filamentous appendages at apex. The basal spine-like appendages arise lateral from the parameral complex, they are connected by a thinly sclerotized sheath. The distal pair is part of the long and thin distiphallus sclerite. This sclerite is basally joined to the middle part of the parameral complex.

*Remarks*: The specimen described is close to the extant group of Old World *Nemopalpus* species, due to wing venation, and the comparatively long palps that reach the fifth flagellomere. The terminalia are of simple construction with well sclerotized parameres. However, if the two filamentous appendages at the apex of the aedeagus were paired penis rods this would be remarkable because all described extant Bruchomyiinae possess only a single phallotrema and no penis rods.

Subfamily: Incertae sedis

*Bamara* **n. gen.**

urn:lsid:zoobank.org:act:B723BE17-64A2-4740-AC58-82E14FD75241.

*Type-species*: *Bamara groehni* n. sp.

*Derivation of name: Bama*, the largest ethnic group in Myanmar.

*Diagnosis*: Psychodids with oval eyes, without eye bridge, 14 flagellomeres, basal segments bottle-shaped, terminal segment reduced in size. Sc short, terminates in R, with crossvein sc-c; R with 4 veins, bases of R_1_ and Rs nearby, R_2_ runs perpendicular to costa. Cell br short, without closed cell bm, with M_3_ and CuA_1_ sharing the same stem; CuA_2_ and anal vein straight. Genitalia inverted, with cerci.

*Remarks*: The combination of oval eyes, 14 flagellomeres, R with 4 branches, Sc short, cell br open and cerci of unusual shape without setae does not meet the definitions of Trichomyiinae or of Psychodinae. *Bamara* shares some morphological characters with *Xenotrichomyia* Azar, Mouawad & Salamé (2015) from the Upper Cretaceous of New Jersey concerning eyes, palpus and radial veins but differs in essential taxonomic characters of the flagellum, wing venation and male terminalia so that a closer relationship is excluded. Furthermore, *Bamara* shows similarities with the genus *Axenotrichomyia* Azar et al. (2015) from Burmese amber concerning number of palpus segments and flagellomeres and the wing venation. *Bamara* differs from *Axenotrichomyia* in the longer anal vein and the shape of male genitalia with cerci elongate and apparently fleshy instead of setose surstyli reduced to a membrane, the presence of a long aedeagal sheath, and the absence of tentacle-like structures on the parameres.

*Bamara groehni* **n. sp.**

urn:lsid:zoobank.org:act:B9FD23CB-8D1B-4F25-AE65-957A16450DE4.

**Figure 16:**
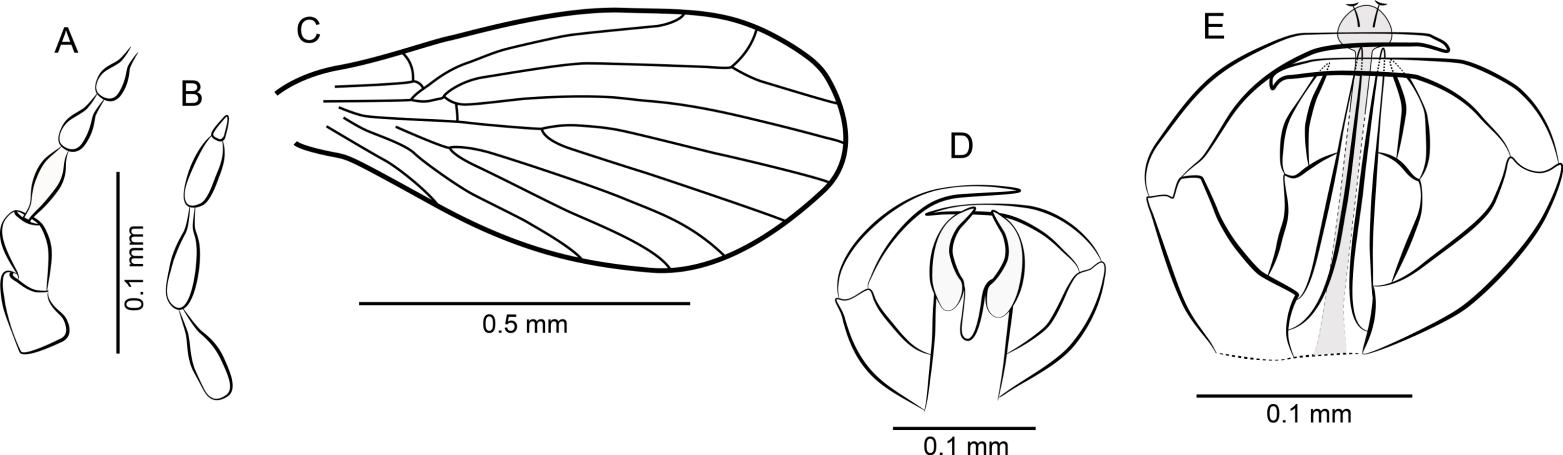
*Bamara groehni* n. sp. ♂. (A) Scape, pedicel and flagellomeres 1–3. (B) Terminal 4 flagellomeres. (C) Wing. (D) Genitalia ventral view. (E) Genitalia dorsal view.

Figs. 16A–16E and 18B.

*Material:* Holotype male GPIH no. 4417, collection Gröhn no. 11006. Syninclusions: Psychodidae, *Parasycorax simplex* n. sp. (GPIH no. 4449, collection Gröhn no. 11020); Psychodidae, *Palaeoparasycorax suppus* n. sp. (GPIH no. 4418, collection Gröhn no. 11006); Psychodidae, *Phlebotomus* sp. (collection Gröhn no. 11019); Psychodidae, Phlebotominae sp. (collection Gröhn no. 11020).

*Type-locality*: Hukawng Valley of the northern state of Kachin.

*Stratigraphic horizon*: 98.79 ± 0.62 million years, Upper Cretaceous (earliest Cenomanian) (Shi et al., 2012).

*Derivation of name*: Dedicated to Carsten Gröhn, who provided specimens for the present scientific study.

*Diagnosis*: A small sparingly hairy psychodid species, with oval eyes; wing elliptic with four radial veins; terminalia inverted, with large and blade shaped parameres.

*Description*: Body length: 0.82 mm.

*Head*: Anterior-posteriorly flat, no eye bridge, vertex high. Eyes oval, situated very laterally on head. Diameter of individual facets about 0.06 mm. Antenna (Figs. 16A and 16B) approximately 0.6 mm long, with 14 flagellomeres. Distal border of scape and basal border of pedicel oblique. Flagellomeres with long hairs, ascoids unrecognizable; flagellomeres 1 to 13 bottle-shaped, slightly decreasing in length, penultimate segment without internode, terminal segment distinct but markedly shorter than the preceding elements, conical and broadly joined to the preceding flagellomere by its entire base. Relative proportions of antennomeres: 10-12-12-10-11-10-9-9-9-9-9-9-10-8-8-2. Mouthparts short, maxillary palpus with 3 short segments.

*Thorax*: Mesonotum with spare hairs.

*Wing*: (Fig. 16C) Approximately 0.85 mm long, width 0.4 mm, scarcely setose, with hairs on veins and along costal vein. Neala not developed. Sc short, ending in radius just after fork R_1_/Rs, with sc-c cross-vein. Radius with 4 veins, all arise from cell br; R_1_ long with its origin from radial stem angulate, radial sector short, as long as sc-c, R_2+3_ long, about as long as R_1_, radial fork (R_2_+R_3_) distal of tip of R_1_, with R_2_ almost perpendicular to costa in that sector. Medial fork (M_1_ + M_2_) just before middle of wing, M_3_ and CuA_1_ sharing a stem; without closed cell bm; anal vein nearly straight.

*Abdomen*: With eight pregenital segments of which six are clearly visible, segment 1 small, segment 3 the largest. Terminalia (Figs. 16D and 16E) inverted by segments 7 and 8 which seem to be partly hidden in segment 6. Terminalia with thin, elongate slightly bent gonocoxites and gonostyli; gonostylus 1.2 times longer than gonocoxite, both without specialized setae or tubercles. Gonocoxites basally with dorsal situated blade shaped appendages (parameres) which are longer than gonocoxites. At centre, the aedeagal sheath ends in a globular tip from which two T-shaped fine sclerites emerge, corresponding probably to the penis (or a pair of penis rods). Epandrium rectangular, longer than wide, basally and distally deeply invaginated. Cerci elongate, slightly bent, flat, held in vertical plane; they appear fleshy and are without specialized setae or tubercles.

*Remarks:* The radius sector is very short and veins R_1_, R_2_ + R_3_ and R_4+5_ originate at short distances in the basal third of wing. The size reduction of terminal abdomen segments and the inversion of the genitalia probably by segments 7 and 8 provides an unusual set of characters.

*Bamara* sp.

**Figure 17:**
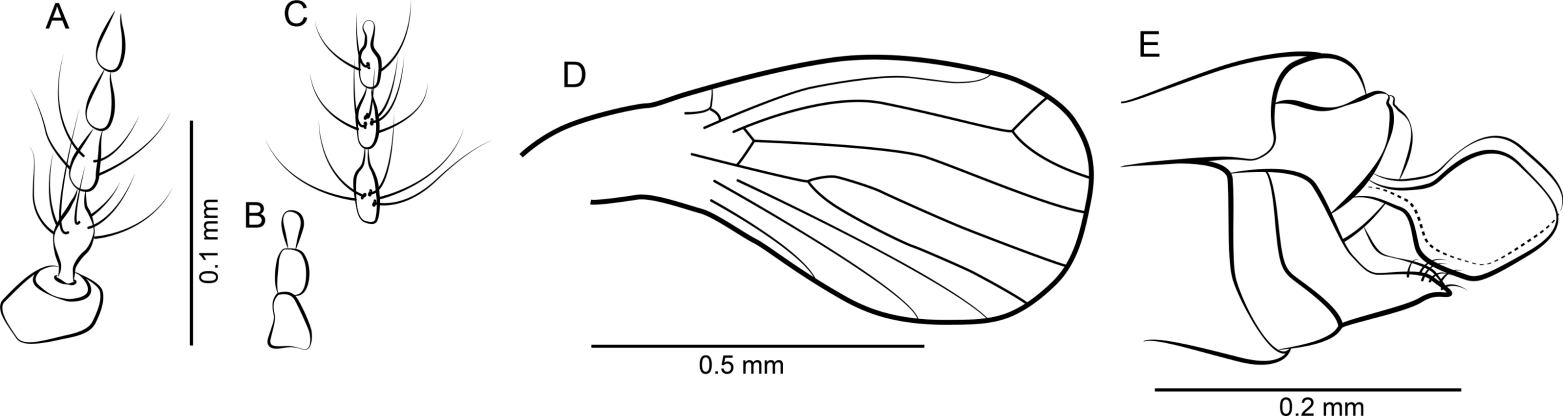
*Bamara* sp. ♀. (A) Pedicel and flagellomeres 1–4. (B) Palpus. (C) Terminal 3 flagellomeres. (D) Wing. (E) Terminalia lateral view.

**Figure 18:**
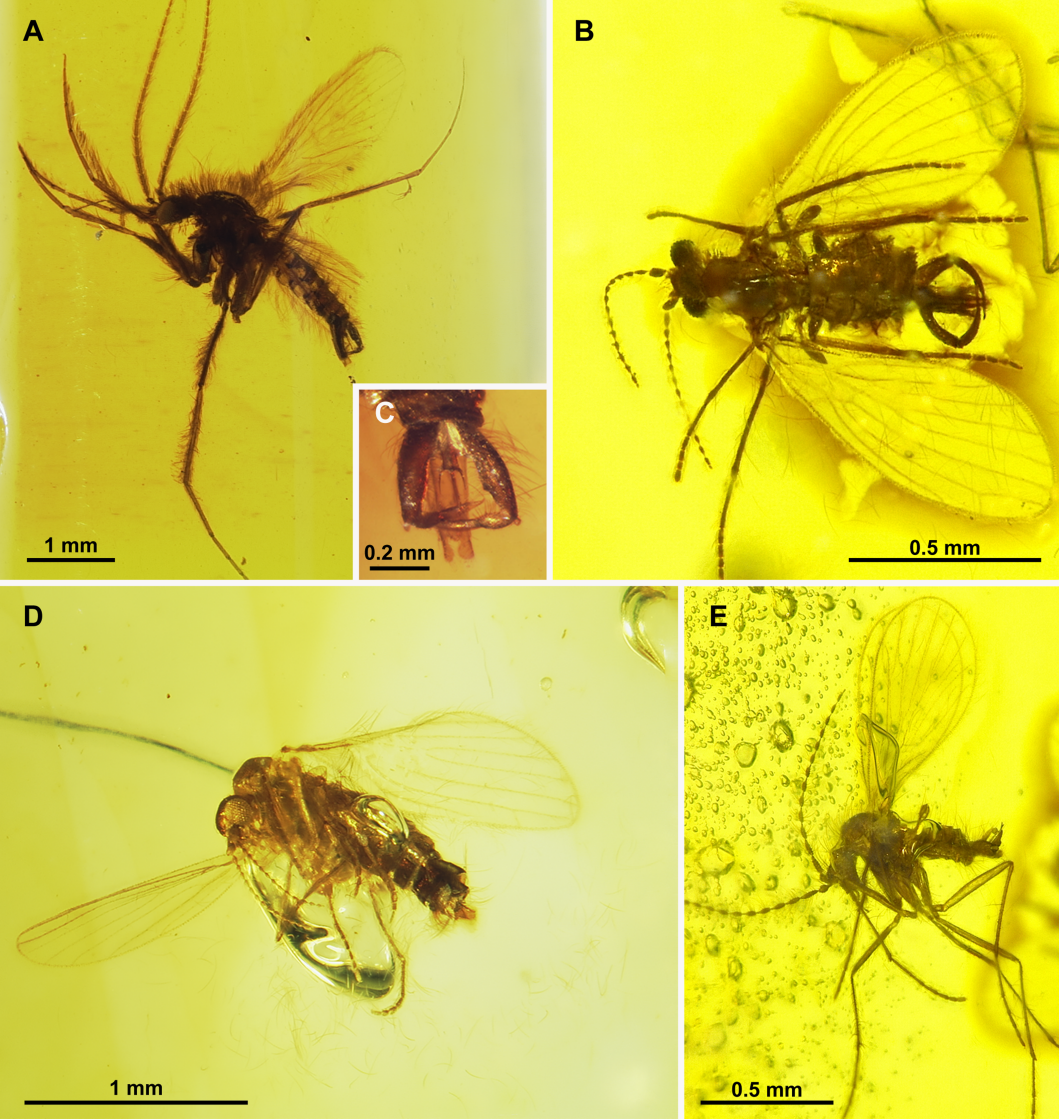
Bruchomyiinae, Sycoracinae and subfamily incertae sedis. (A) *Nemopalpus quadrispiculatus* n. sp. ♂, Holotype specimen. (B) *Bamara groehni* n. sp. ♂, Holotype specimen. (C) *Nemopalpus quadrispiculatus* n. sp. ♂, Holotype: terminalia dorsal view. (D) *Bamara* sp. ♀. (E) *Palaeoparasycorax suppus* n. sp. ♂, Holotype specimen.

Figs. 17A–17E and 18D.

*Material:* Female SMF Be 2383. Syninclusion: Sternorrhyncha (1).

*Locality*: Hukawng Valley of the northern state of Kachin.

*Stratigraphic horizon*: 98.79 ± 0.62 million years, Upper Cretaceous (earliest Cenomanian) (Shi et al., 2012).

*Description*: Body length without head: 0.95 mm.

*Head*: In ventro-lateral view wider than long, no eye bridge, vertex high. Eyes oval, situated laterally on head. Antenna (Figs. 17A and 17C) approximately 0.5 mm long, with 13 flagellomeres. Scape cylindrical, pedicel bulbous. Flagellomeres with long hairs, ascoids unrecognizable; flagellar segments 1 to 12 bottle-shape, slightly decreasing in length, terminal segment with apiculus. approximate relative proportions of antennomeres (as articles are not arranged in a single plane): 10-11-14-9-10-9-9-10-11-10-11-10-11-9-11. Mouthparts short, probably not functional, palpus (Fig. 17B) short, with 3 segments, basal segment the longest. Relative proportions of articles: 10-7-6.

*Thorax*: Mesonotum with sparse hairs.

*Wing*: (Fig. 17D) Approximately 1.1 mm long, scarcely setose, with hairs on veins and along costal vein. Veins in the basal part of the wing not recognizable. Sc terminates in radius, with sc-c cross-vein. Radius with 4 veins; R_1_ and R_2+3_ long, fork R_2_ + R_3_ at about the level of tip of R_1_, R_2_ runs almost perpendicular to costa in this sector. Fork M_1_/M_2_ in the basal half of wing.

*Abdomen*: With eight clearly visible segments. Cerci (Fig. 17E) with a thin basal peduncle and a distal sclerotized square-shaped part. Subgenital plate setose, triangular with acute apex.

*Remarks*: Due to the superficial similarity in wing venation and number of palpomeres the specimen is included in *Bamara*.

## Discussion

### Protopsychodinae

Specimens of Protopsychodinae do not fit into the concept of extant subfamilies of Psychodidae. The combination of characters such as antenna with 14 flagellomeres, palpus with 5 segments and the last one elongate, wing with humeral crossvein, five R branches, three medial, two CuA and one long almost straight anal veins, with two closed cells br and bm, quadrate rigid epandrium, and surstyli instead of cerci was unknown so far. Protopsychodinae are represented by at least two different genera in Burmese amber: *Datzia* n. gen. and *Mandalayia* n. gen. These genera can be distinguished by the more slender shape of the *Mandalayia* wing, the converging tips of CuA_2_ and A_1_ veins in *Datzia* whereas both run almost parallel to the wing margin in *Mandalayia*. It remains debatable whether the appendages of male tergum 9 (epandrium) should be termed cerci or surstyli, the general shape, however, resembles cerci, but the stiffness and the presence of strong and modified setae in *D. bispina* n. sp. may indicate surstyli. Female genitalia of *Datzia* are with ‘soft’ but not with heavily sclerotized cerci as in extant Psychodinae.

Twelve taxa of Psychodidae (4 phlebotomid flies and 8 psychodid flies) and 2 taxa of an incertae sedis psychodid family (*Eophlebotomus gezei* and *Xenopsychoda harbi*) have been studied and described from the Lower Cretaceous Lebanese amber (Hennig, 1972; Azar et al., 1999; Azar & Nel, 2002; Azar et al., 2003; Azar & Ziadé, 2005). The 8 psychodid taxa belong to 5 genera (*Paleopsychoda*, *Protopsychoda*, *Libanopsychoda*, *Cretapsychoda* and *Paralibanopsychoda*) of the subfamily Psychodinae. Unlike modern Psychodinae, species of *Paleopsychoda* have elongate mouthparts as they occur in representatives of Protopsychodinae. Furthermore *Paleopsychoda* and Protopsychodinae genera share the following set of characters: antenna with 14 flagellomeres, R with 5 branches, a very long anal vein, inverted male genitalia (although the segments involved in inversion remain unclear in *Paleopsychoda*). However, in contrast to *Paleopsychoda,* Protopsychodinae representatives have round eyes without eye bridge, 5 palpomeres instead of 4, and CuA_2_ and A veins terminating in costa very close to one another. Moreover, Protopsychodinae male surstyli are elongate with strong specialized setae (in *Datzia*) whereas males of *Paleopsychoda* have short and setose cerci.

*Xenopsychoda harbi* which is attributed to an incertae sedis family of Psychodoidea by Azar & Ziadé (2005) shares the following characters with representatives of Protopsychodinae: antenna with 14 flagellomeres (15 in Azar & Ziadé (2005) because they count the drop-shaped apiculus of the 14th antennomere as a flagellomere), elongate mouthparts, wing oval, R with 5 branches, thus 2 veins between radial and medial forks, R_5_ originates distal to the R_2+3_ + R_4_ fork, Sc and A veins elongate, CuA_2_ and A veins terminate in costa close to one another. It differs from known species of Protopsychodinae in the number of palpomeres which is 4 in *Xenopsychoda* and 5 in Protopsychodinae, in the presence of a well-developed eye bridge in *Xenopsychoda* while all known species of Protopsychodinae have round eyes without bridge or with very weak developed eye bridge, and in the presence of a crossvein between M_2_ and M_3_. Since *Xenopsychoda* is known from a single female fossil only, the structure of the male terminalia—i.e., if they are of the inverted type and possess elongate cercopodia as male species of Protopsychodinae—remains unclear.

### Sycoracinae

Wing venation with R with 5 branches, i.e., R_4_ and R_5_ joined to a single vein (R_4+5_) occurs in extant Psychodidae only in subfamilies Sycoracinae and Trichomyiinae.

It is difficult to attribute *Palaeoparasycorax* n. gen. clearly to one of the aforementioned subfamilies, and this partly depends on the fact that the subfamily once named Trichomyiinae has been split into Trichomyiinae and Sycoracinae, and that both these subfamilies urgently require revision. However, it seems to be more closely related to Sycoracinae than to Trichomyiinae; the reasons are sc terminates in acute angle in R_1_ and the general shape of the flagellomeres with no or very small ascoids. Not typical in Sycoracinae is the terminal palpomere that is exceptionally long and seemingly slightly sclerotized and apically dilated. A proper classification may be possible after revisions of both groups, including amber species.

### Phlebotominae

Two specimens described here can easily be assigned to Phlebotominae due to elongate mouthparts, antennae features, and male terminalia with lateral prolongations of tergum 9 and several remarkably long and strong bristles on the gonostyli.

### Bruchomyiinae

*Nemopalpus quadrispiculatus* can easily be assigned to Bruchomyiinae, based on wing venation, structure and length relation of antennae and mouthparts, and male terminalia. Palpi are comparatively long, the tips reaching flagellomere 6. Gonocoxites and gonostyli are of simple construction and the parameres are sclerotized with two elongate terminal prolongations. If the two distal prolongations are paired penis rods this must be interpreted as plesiomorphy, because all described extant Bruchomyiinae seem to possess only a single gonoporus.

### Subfamily incertae sedis

*Bamara* shows a unique set of characters which does not meet the definitions of Trichomyiinae or of Psychodinae, and representatives of this genus may belong to another undescribed Psychodidae subfamily.

## Conclusions

Having inverted terminalia with torsion only by segment 9 with a basal ring—which otherwise only occurs in extant Psychodinae—Protopsychodinae probably represent the so far oldest known relative of modern Psychodinae. Furthermore, in Protopsychodinae the epandrium has the form of a plate and there are surstyli (epandrial articulated lobes which can have modified setae)—both characteristics which occur in Psychodinae—but the palpus is 5-segmented and the eyes rounded as in Bruchomyiinae and Phlebotominae. Bruchomyiinae have a well-developed epandrium but in Phlebotominae it is reduced and membranous. This combination of characteristics may demonstrate the monophyly of Psychodidae, a question that still is in doubt for some dipterologists (e.g., Abonnenc & Léger, 1976; Lewis et al., 1977).

Findings of two pieces of amber each containing numerous male induviduals of *Datzia bispina* (8♂, 6♂) indicate mating swarm behaviour of this group. Moreover, Protopsychodinae possess elongate mouthparts, supporting the presumption that at least females of this subfamily were blood-feeders. Besides this group only Phlebotominae and Sycoracinae feed on blood, both of which are reported here from Burmese amber. Including the knowledge of all other described fossil blood-feeding Psychodidae it can be concluded that diversity of these taxa was high in the Cretaceous and sufficient prey must have been available.

The assignment of *Palaeoparasycorax* to Sycoracinae remains difficult until there is a stable classification of this subfamily. Revision of this subfamily however is outside the scope of the present work. Likewise, the systematic position of *Bamara* remains unclear at this moment.

## Additional Information and Declarations

### New Species Registration

The following information was supplied regarding the registration of a newly described species: New subfamily Protopsychodinae: LSID urn:lsid:zoobank.org:act:568EDF6C-C7B1-457D-AF44-4C4D643C00B6 New genera *Bamara*: LSID urn:lsid:zoobank.org:act:B723BE17-64A2-4740-AC58-82E14FD75241 *Datzia*: LSID urn:lsid:zoobank.org:act:A8D8B8AE-229E-469F-A8F8-CA496B45FFA0 *Mandalayia*: LSID urn:lsid:zoobank.org:act:7190BF4B-65D9-46DD-8707-0817D481E6F6 *Palaeoparasycorax*: LSID urn:lsid:zoobank.org:act:B4539700-0E5D-412B-A345-AD48E5EEBB99 New species *Bamara groehni*: LSID urn:lsid:zoobank.org:act:B9FD23CB-8D1B-4F25-AE65-957A16450DE4 *Datzia bispina*: LSID urn:lsid:zoobank.org:act:B094011C-331E-487E-8700-B4FB53EEB95C *Datzia setosa*: LSID urn:lsid:zoobank.org:act:5C8AEAFA-607B-491A-A31C-3B906B57111F *Mandalayia beumersorum*: LSID urn:lsid:zoobank.org:act:575A0DF6-8F3C-434B-8264-67C87874727D *Nemopalpus quadrispiculatus*: LSID urn:lsid:zoobank.org:act:EB83F66F-4660-45E3-9F79-A04AF27FEAB7 *Palaeoparasycorax globosus*: LSID urn:lsid:zoobank.org:act:18F913A7-6A8D-4E43-924B-E41392850903 *Palaeoparasycorax suppus*: LSID urn:lsid:zoobank.org:act:6CB43B4E-83D5-42B3-ACE4-0D809C79FA08 *Parasycorax simplex*: LSID urn:lsid:zoobank.org:act:43D74B77-031E-435F-9FF9-AA25C1BFBEC1 *Phlebotomites aphoe*: LSID urn:lsid:zoobank.org:act:BFE03C00-720A-4186-BD34-646D9B8BF7F4 *Phlebotomus vetus*: LSID urn:lsid:zoobank.org:act:4217A01A-AB5B-47D0-845B-DFF9957390B6.
